# Supplementary material for: m6A modification of circHPS5 and hepatocellular carcinoma progression through HMGA2 expression
Source: Mol Ther Nucleic Acids. 2021 Sep 14;26:637–48. doi: 10.1016/j.omtn.2021.09.001 (PMC8517093; doi:10.1016/j.omtn.2021.09.001)
Supplement: Document S1. Tables S1–S2 and Figures S1–S5 [file mmc1.pdf]

OMTN, Volume *26*

Supplemental information

m6A modification of circHPS5 and hepatocellular carcinoma progression through HMGA2 expression

Dawei Rong, Fan Wu, Chen Lu, Guangshun Sun, Xiaoli Shi, Xiaoyuan Chen, Yongjiu Dai, Weizhe Zhong, Xiaopei Hao, Jinren Zhou, Yongxiang Xia, Weiwei Tang, and Xuehao Wang

Supplementary Table 1 Analysis between values and expression level of circHPS5 in HCC

Parameters	Low N=13	High N=33	P-value
**Age(years)**			0.463
＜ 60	8	18	
≥ 60	5	15	
**Gender**			0.305
Male	10	29	
Female	3	4	
**Size(cm)**			0.000*
＜ 5	13	8	
≥ 5	0	25	
**TNM stage**			0.006*
I	12	16	
II–IV	1	17	
**Microvascular invasion**			0.015*
Negative	12	18	
Positive	1	15	
**Virus infection**			0.330
Negative	2	9	
Positive	11	24	

* means P＜0.05

Supplementary　Table 2 sequence of all genes and probes

name	sequence
circHPS5-F	TCTGCAACCAGAGTCTTTAAGGT
circHPS5-R	GGTTCAGTCGTGTGTTACGA
HPS5-F	TCGATCCTTCTTGTGTGACACT
HPS5-R	GGTGGCAACGAGAGGAGTTTC
GAPDH-F	GGGAAACTGTGGCGTGAT
GAPDH-R	GAGTGGGTGTCGCTGTTGA
RARA-F	GAAGTGCTTTGAAGTGGGCA
RARA-R	GTCCCAGAGGTCAATGTCCA
miR-1183-F	ACTGACCACTGTAGGTGATGGT
miR-1183-R	GCGAGCACAGAATTAATACGACTCACTATAGG
miR-1299-F	ACACTCCAGCTGGGTTCTGGAAUUCTC
miR-1299-R	CAGTGCGTGTCGTGGAGT
miR-370-3p-RT	GTCGTATCCAGTGCAGGGTCCGAGGTATTCGCACTGGATACGACACCAGG
miR-370-3p-F	GCCTGCTGGGGTGGAACC
miR-370-3p-R	CAGTGCAGGGTCCGAGGT
U6-F	GCTTCGGCAGCACATATACTAAAA
U6-R	CGCTTCACGAATTTGCGTGTCAT
sh-circHPS5-1#	AAGGTTATCCCAGTGAGCATT
sh-circHPS5-2#	GAAGGTTATCCCAGTGAGCAT
sh-circHPS5-3#	GGTTATCCCAGTGAGCATTGT
si-METTL3	GCACAUCCUACUCUUGUAATT
si-YTHDC1	GCAAGGAGUGUUAUCUUAATT

control-probe	UUGUACUACACAAAAGUACUG
circHPS5-probe	ACACAATGCTCACTGGGATAACCTTCATC
miR-370-probe	ACCAGGTTCCACCCCAGCAGGC

Figure S1 Agarose gel electrophoresis was employed for the verification of circHPS5 specificity.

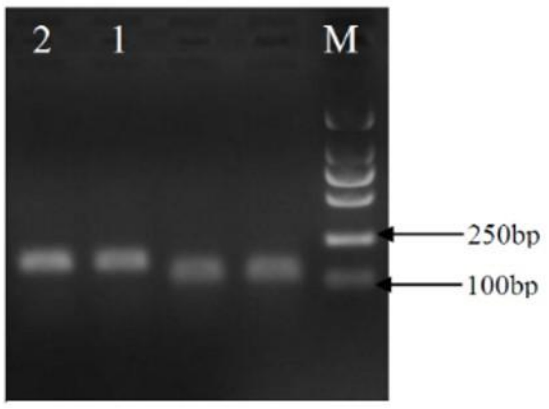

Figure S2 (a) Over-expression of circHPS5 was designed to up-regulate circHPS5 level in HCC cells. (b)The growth curves of cells were measured after transfection with vector and circHPS5 by using CCK-8 assays. (c)Cloning formation assay was performed to evaluate cell proliferation.(d)EdU assays of HCC cells transfected with vector and circHPS5 were performed to evaluate cell proliferation. (e) Flow cytometry was used to assess cell apoptosis.(f)Transwell experiment was used to assess cell invasion.(g) Cell motility was examined in cells transfected with vector or circHPS5 by by wound healing assay. *p < 0.05,**p < 0.01,***p < 0.001.

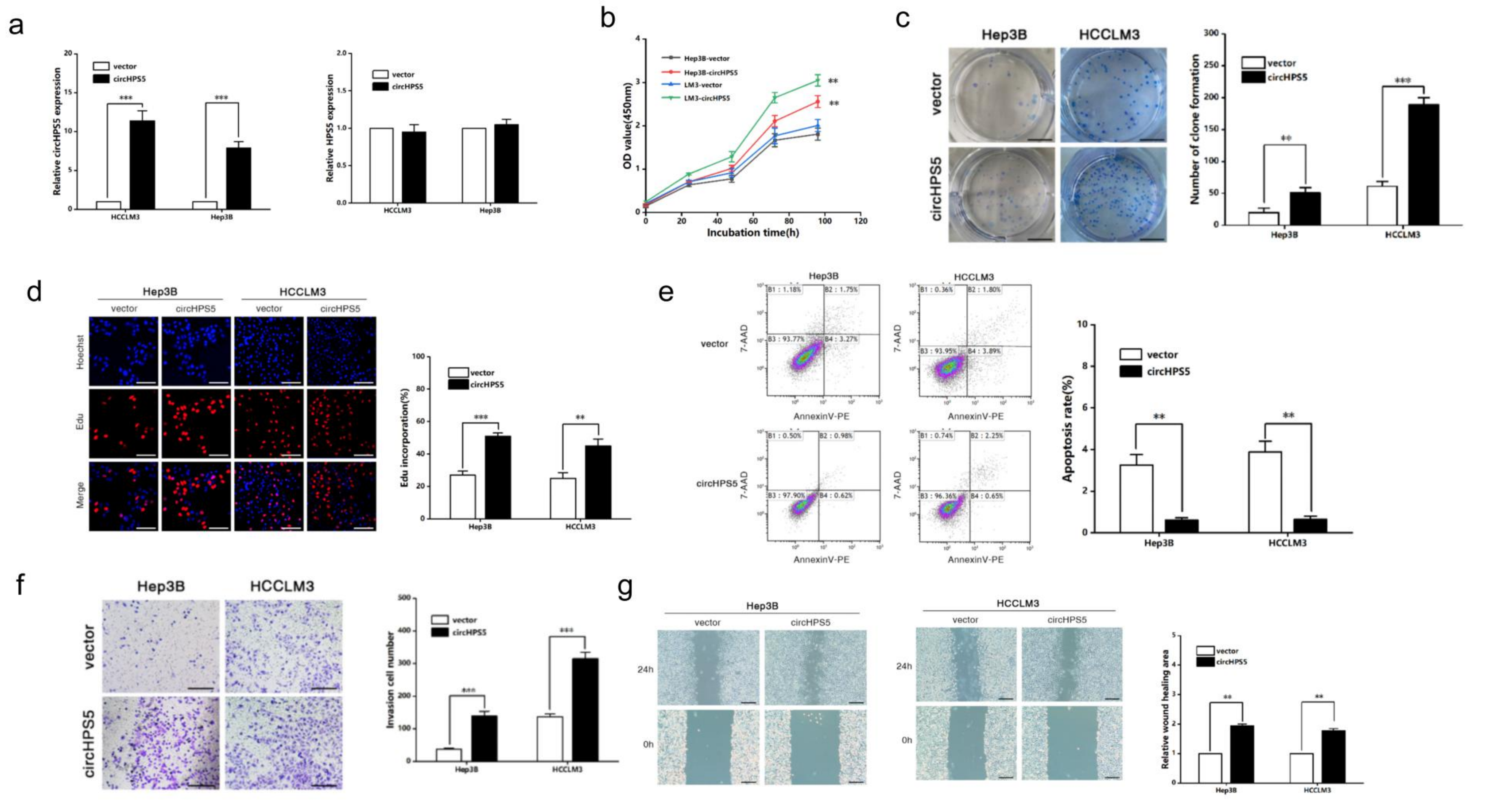

Figure S3(a)Immunofluorescence was employed to detect the expression of E-cadherin, Vimentin ,and α-actin . (b)Morphological changes of EMT in cancer cells. (c)EMT related protein expression in ector and circHPS5 group.(d) Morphological changes of CSC in cancer cells.(e) CSC related markers expression in vector or circHPS5 group. *p < 0.05,**p < 0.01.

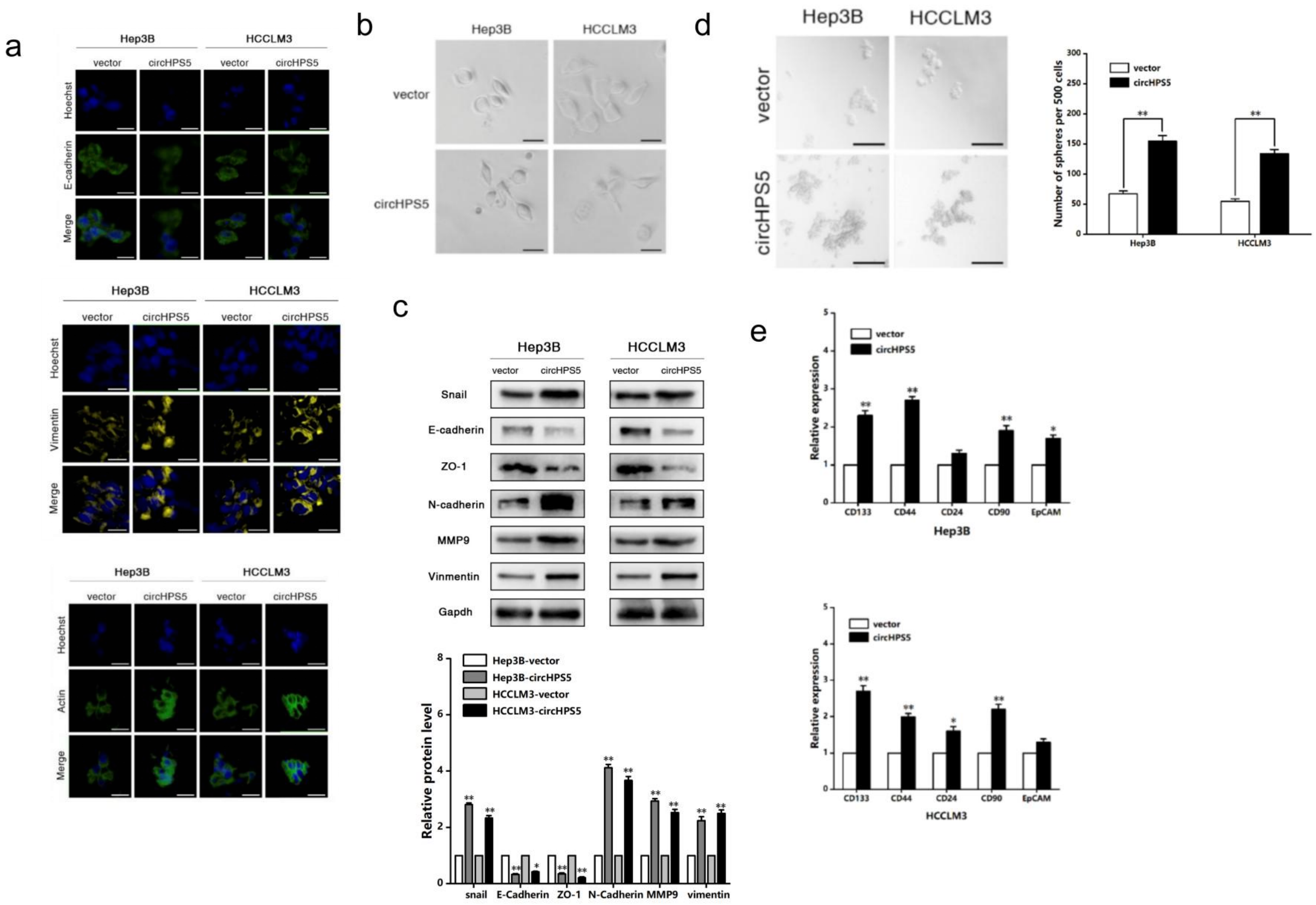

Figure S4(a) Flow chart of methylation sequencing.(b)The expression distribution map describes the normalized expression distribution intensity and abundance after the sequence is aligned to the genome.(c)The sample correlation diagram proves that the involved biological experiment operations can be repeated with little variation, ensuring that the subsequent differential gene analysis can get more reliable results.(d)Peak distribution pie chart on RNA structure.(e)We used HOMER software to perform motif analysis on peaks and found that the typical motif modified by m6A is "GGAC".(f)SRAMP prediction website revealed that circHPS5 was highly m6A modified.

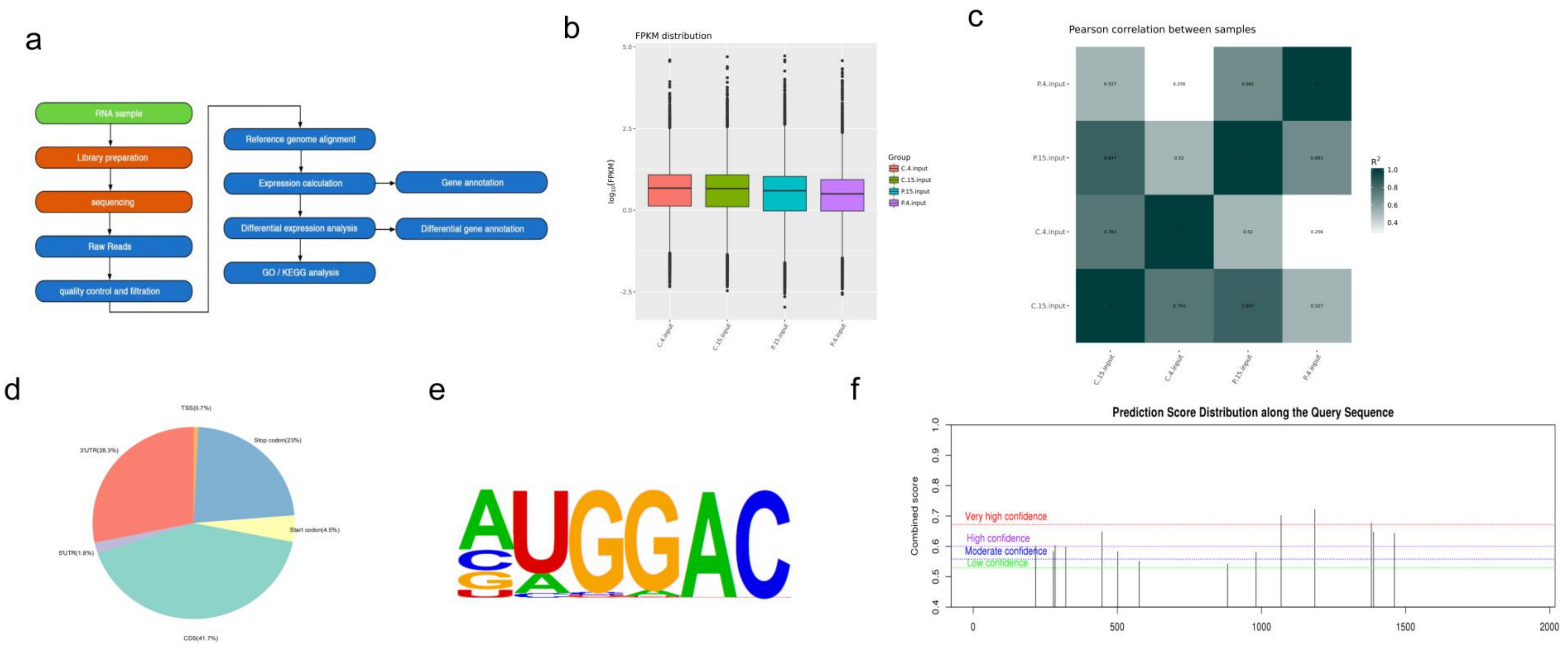

Figure S5 (a) Morphological changes of EMT in cancer cells in different groups. (b)Immunofluorescence was employed to detect the expression of E-cadherin, Vimentin ,and α-actin in different groups.(c)Morphological changes of CSC in cancer cells.(d) CSC related markers expression in different groups. $^*p < 0.05$,$^{**}p < 0.01$,$^{***}p < 0.001$.

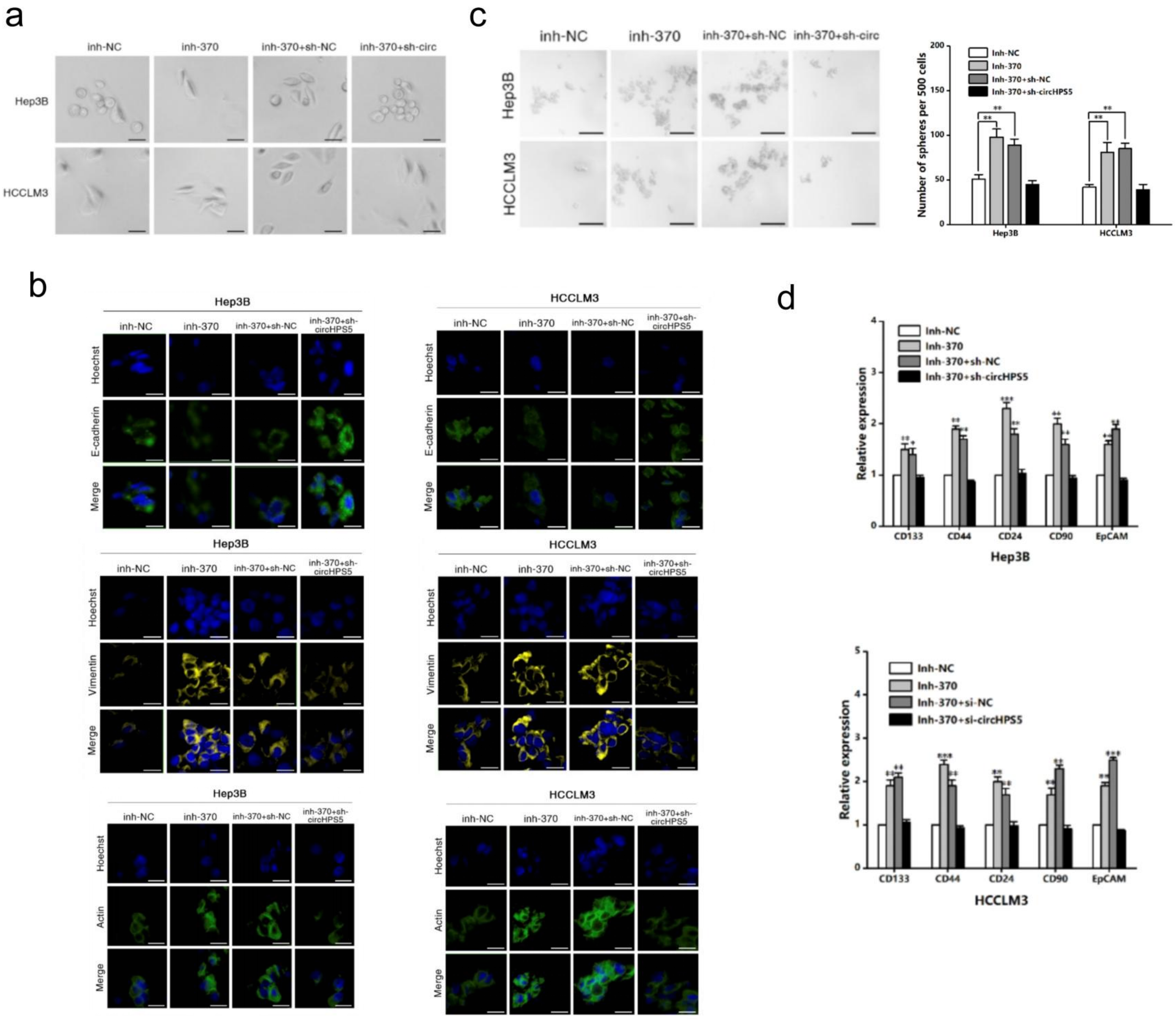
a
inh-NC
inh-370
inh-370+sh-NC
inh-370+sh-circ
Hep3B
HCCLM3
c
inh-NC
inh-370
inh-370+sh-NC
inh-370+sh-circ
Hep3B
HCCLM3
Inh-NC
Inh-370
Inh-370+sh-NC
Inh-370+sh-circHPS5
Number of spheres per 500 cells
200
150
100
50
0
**
Hep3B
HCCLM3
b
Hep3B
HCCLM3
inh-NC
inh-370
inh-370+sh-NC
inh-370+sh-circHPS5
Hoechst
E-cadherin
Merge
Vimentin
Actin
d
Inh-NC
Inh-370
Inh-370+sh-NC
Inh-370+sh-circHPS5
Relative expression
CD133
CD44
CD24
CD90
EpCAM
Hep3B
Inh-370+si-NC
Inh-370+si-circHPS5
HCCLM3
