# Supplementary material for: m6A modification of circHPS5 and hepatocellular carcinoma progression through HMGA2 expression
Source: Mol Ther Nucleic Acids. 2021 Sep 14;26:637–48. doi: 10.1016/j.omtn.2021.09.001 (PMC8517093; doi:10.1016/j.omtn.2021.09.001)
Supplement: Document S2. Article plus supplemental information [file mmc2.pdf]

Molecular Therapy
Nucleic Acids
Original Article

m6A modification of circHPS5 and hepatocellular carcinoma progression through HMGA2 expression

Dawei Rong,[1,2,5] Fan Wu,[3,5] Chen Lu,[4,5] Guangshun Sun,[3] Xiaoli Shi,[1,2] Xiaoyuan Chen,[1,2] Yongjiu Dai,[1] Weizhe Zhong,[1] Xiaopei Hao,[1] Jinren Zhou,[1] Yongxiang Xia,[1] Weiwei Tang,[1] and Xuehao Wang[1]

[1]Hepatobiliary/Liver Transplantation Center, The First Affiliated Hospital of Nanjing Medical University, Key Laboratory of Living Donor Transplantation, Chinese Academy of Medical Sciences, Nanjing, Jiangsu, China; [2]School of Medicine, Southeast University, Nanjing, Jiangsu, China; [3]Department of General Surgery, Nanjing First Hospital, Nanjing Medical University, Nanjing, Jiangsu, China; [4]Department of General Surgery, The First Affiliated Hospital of Nanjing Medical University, Nanjing, Jiangsu, China

N6-methyladenosine (m6A) is capable of mediating circRNA generation in carcinoma biology. Nevertheless, the posttranscriptional systems of m6A and circRNA in hepatocellular carcinoma (HCC) development are still unclear. The present study identified a circRNA with m6A modification, circHPS5, which was increased in neoplasm HCC tissues and indicated poor patient survival. Silencing of circHPS5 inhibited epithelial-mesenchymal transition (EMT) and cancer stem-like cell (CSC) phenotypes. Notably, METTL3 could direct the formation of circHPS5, and specific m6A controlled the accumulation of circHPS5. YTHDC1 facilitated the cytoplasmic output of circHPS5 under m6A modification. In addition, we demonstrated that circHPS5 can act as a miR-370 sponge to regulate the expression of HMGA2 and further accelerate HCC cell tumorigenesis. Accordingly, the m6A modification of circHPS5 was found to modulate cytoplasmic output and increase HMGA2 expression to facilitate HCC development. The new regulatory model of "circHPS5-HMGA2" provides a new perspective for circHPS5 as an important prognostic marker and therapeutic target in HCC and provides mechanistic insight for exploring the carcinogenic mechanism of circHPS5 in HCC.

INTRODUCTION

Liver carcinoma is the most common carcinoma worldwide, and carcinoma statistics in 2018 show that the global incidence of liver carcinoma ranks sixth and the mortality rate ranks fourth.[1] Due to hepatitis B virus (HBV) infection, most cases (80%) occur in East Asia and Sub-Saharan Africa.[2] Hepatocellular carcinoma (HCC) accounts for > 80% of primary liver carcinoma cases worldwide.[3] Risk elements for HCC consist of exposure to dietary toxins (e.g., aflatoxins and aristolochic acid), metabolic liver disease (e.g., nonalcoholic fatty liver disease), alcohol addiction, and chronic hepatitis B and C.[4] However, HCC has the typical characteristics of high malignancy—difficult treatment, rapid development, and short survival time. Therefore, studying HCC occurrence and development is of great significance to prolong the survival of HCC patients.

N6-methyladenosine (m6A) describes a methylation process at the N6 position of adenosine and is the most abundant inner modifying process in eukaryote mRNA.[5] After it was initially identified in 1974, studies of m6A boomed due to advancements in detection approaches and the identification of vital regulating proteins. In addition, m6A-modification processes were suggested to control the generating processes and functions of transfer RNA (tRNA), ribosomal RNA (rRNA) and noncoding RNAs (ncRNAs) (including circular RNAs (circRNAs), long noncoding RNAs (lncRNAs), and microRNA (miRNA)).[5–7] circRNAs, a novel endogenous noncoding RNA class, were found in the early 1990s to be transcripts exhibiting a scrambled exon order; furthermore, their structure, system and function were identified, and they have become research hotspots in the past 20 years.[8,9] According to Yang et al., m6A modification of circRNAs can produce inner ribosomal entry sites (IRESs) to translate cap-independent proteins. This m6A-promoted process to translate circRNAs can be improved by METTL14 and METTL3, and the m6A demethylase FTO-mediated demethylating process is likely to limit this translation procedure. The initiating element, eIF4G2, for the translation process and the m6A reading protein, YTHDF3, are needed.[10] Nevertheless, the systems of the post-transcriptional process of m6A modification and the role of circRNA in carcinoma development are still unclear.

The present study identified a circRNA with the m6A modification, circHPS5, which shows a frequent increase in neoplasm tissues

Received 24 June 2021; accepted 8 September 2021;
https://doi.org/10.1016/j.omtn.2021.09.001.

[5]Senior author

Correspondence: Yongxiang Xia, Hepatobiliary/Liver Transplantation Center, The First Affiliated Hospital of Nanjing Medical University, Key Laboratory of Living Donor Transplantation, Chinese Academy of Medical Sciences, Nanjing, Jiangsu, China.
E-mail: yx_xia@njmu.edu.cn
Correspondence: Weiwei Tang, Hepatobiliary/Liver Transplantation Center, The First Affiliated Hospital of Nanjing Medical University, Key Laboratory of Living Donor Transplantation, Chinese Academy of Medical Sciences, Nanjing, Jiangsu, China.
E-mail: 1243773473twww@sina.com
Correspondence: Xuehao Wang, Hepatobiliary/Liver Transplantation Center, The First Affiliated Hospital of Nanjing Medical University, Key Laboratory of Living Donor Transplantation, Chinese Academy of Medical Sciences, Nanjing, Jiangsu, China.
E-mail: wangxh@njmu.edu.cn

This is an open access article under the CC BY license (http://creativecommons.org/licenses/by/4.0/).

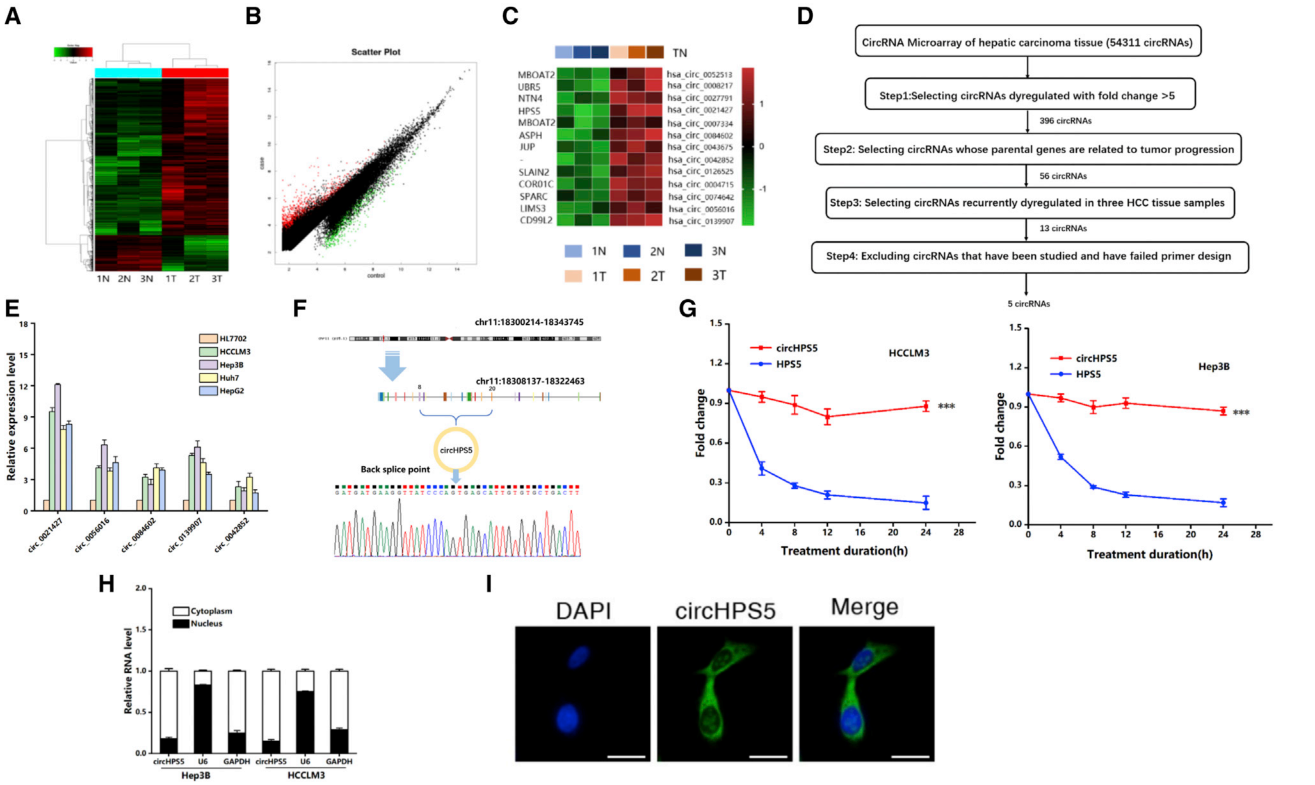

Figure 1. Characterization and expression of circHPS5 in HCC

(A and B) Cluster map and scatterplot of circRNA microarray data showing differentially expressed circRNAs in the HCC group and normal group. A high expression level is indicated by ''red'' and a low expression level by ''green''. (C and D) Flowchart illustrating the screening criteria for potential regulatory circRNAs enriched in HCC. Clustered heatmap showing the dysregulated expression of circRNAs in three HCC samples (the laverage normalized fold changel > 5). (E) The expression of five circRNAs was evaluated in HCC cell lines using qRT-PCR. (F) The genomic locus of circHPS5. The expression of circHPS5 was detected by qRT–PCR followed by Sanger sequencing. (G) qRT-PCR analysis to determine the abundance of circHPS5 and HPS5 mRNA in HCC cells treated with actinomycin. (H) Nuclear and cytoplasmic separation experiments showing the distribution of circHPS5. U6 was used as a marker to show efficient nuclear/cytoplasmic RNA separation. (I) RNA fluorescence *in situ* hybridization for circHPS5. Nuclei were stained with DAPI. Scale bar, 10 μm. ***p < 0.001.

from HCC cases and predicts poor patient survival. circHPS5 overexpression can facilitate epithelial-mesenchymal transition (EMT) and cancer stem-like cell (CSC) phenotypes, further promoting HCC migration and proliferation *in vitro* and *in vivo*. However, knockdown of circHPS5 leads to the opposite results. Notably, YTHDC1 facilitates the cytoplasmic output of circHPS5 under m6A modification. In addition, we demonstrate that circHPS5 can act as a miR-370 sponge to regulate HMGA2 expression and further accelerate HCC cell development.

RESULTS

Characterization of circHPS5 in HCC

This study evaluated a human circRNA microarray with significant throughput based on carcinoma tissues and paracancerous normal tissues from three HCC cases. As shown in Figures 1A and 1B, a cluster map and scatterplot show that the HCC group had 5,121 circRNAs with differential expression compared with the control group: 4,161 upregulated and 960 downregulated circRNAs. From these differentially expressed circRNAs, based on a fold difference greater than 5, circRNA characteristics, and parental genes, we finally identified a total of five target circRNAs (circ_0021427,circ_0056016,circ_0084602, circ_0139907,circ_0042852) for in-depth verification and analysis (Figures 1C and 1D). The expression of these five circRNAs in normal hepatocytes and HCC cells was explored via quantitative real-time PCR (qRT-PCR). As revealed from the results, these five circRNAs were upregulated in HCC cells compared with normal hepatocytes. Among them, circHSP5 (circ_0021427) from the HPS5 gene exhibited the highest upregulation (Figure 1E). The spliced mature sequence of circHPS5, located in chr11:18308137-18322463, is 1941 bp in length, in accordance with the circBase information database (http://www.circbase.org/), and circHPS5 is derived from exons 8–20 (Figure 1F). Moreover, Sanger sequencing results were consistent with the base sequence of circHPS5 (Figure 1F). Resistance to digestion with actinomycin further confirmed that this RNA species was circular in form (Figure 1G). Agarose gel electrophoresis was employed to verify circHPS5 specificity (Figure S1). According to nuclear and cytoplasmic separation, circHPS5 exhibited a distribution in both the nucleus and cytoplasm, with a higher distribution in the cytoplasm than

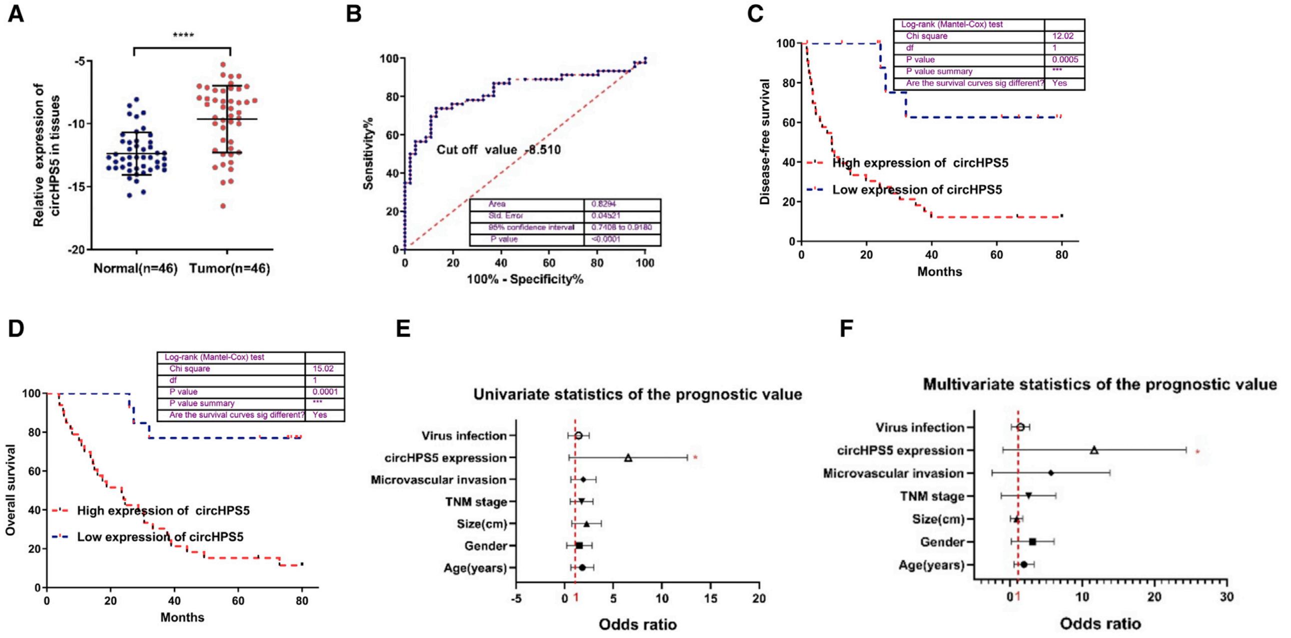

Figure 2. circHPS5 is significantly upregulated in HCC and associated with a poor prognosis

(A) circHPS5 expression was measured using qRT-PCR in 46 pairs of HCC (tumor) and matched noncancerous (normal) tissues. (B) The AUC in distinguishing HCC tissues and normal tissues. (C) and (D) Kaplan–Meier survival curve showing the relationship between circHPS5 and disease-free survival and overall survival time. (E) Univariate and (F) multivariate regression analyses of factors affecting the prognosis of HCC. *p < 0.05, ***p < 0.001, ****p < 0.0001.

in the nucleus (Figure 1H), which was further verified by *in situ* RNA hybridization (FISH) (Figure 1I).

circHPS5 is significantly upregulated in HCC and associated with a poor prognosis

We detected the expression level of circHPS5 in 46 paired HCC tissues and normal tissues adjacent to carcinoma using qRT–PCR and found that circHPS5 was significantly overexpressed in HCC tissues compared with paracancerous tissues (Figure 2A). Clinicopathological features showed that circHPS5 overexpression was positively associated with neoplasm size, tumor lymph node metastasis (TNM) stage, and microvascular invasion (Table S1). Furthermore, the area under the ROC curve (AUC) of circHPS5 in distinguishing HCC tissues and normal tissues was 0.8294, and the cut-off value was −8.510 (Figure 2B). According to a Kaplan–Meier survival curve, patients with higher circHPS5 expression showed reduced disease-free survival and overall survival times (Figures 2C and 2D). Univariate and multivariate analyses indicated that high circHPS5 expression could serve as an independent prognostic indicator for the overall survival of HCC patients (Figures 2E and 2F).

circHPS5 plays a promoting role in HCC *in vitro*

To assess the effect exerted by circHPS5 in HCC cells, three shRNAs against circHPS5 (sh-circHPS5) were developed to silence circHPS5 with no impact on HPS5 mRNA levels in Hep3B and HCCLM3 cells (Figure 3A). Finally, sh-circHPS5 #1 was used to perform subsequent experiments due to its significant inhibition efficiency (Figure 3A). sh-circHPS5 was capable of inhibiting cell proliferation in Hep3B and HCCLM3 cells, as revealed by the results of CCK-8, colony formation, and 5-ethynyl-20-deoxyuridine (EdU) experiments (Figures 3B–3D). Flow cytometry showed that knockdown of circHPS5 enhanced cancer cell apoptosis (Figure 3E). Transwell and wound healing experiments showed that silencing circHPS5 significantly decreased Hep3B and HCCLM3 cell invasion and migration (Figures 3F and 3G), while overexpression of circHPS5 played the opposite role (Figure S2). Immunofluorescence results showed that E-cadherin was upregulated, vimentin was downregulated, and pseudopodia composed of α-actin were decreased, suggesting that the downregulation of circHPS5 can inhibit the EMT phenotype (Figures 4A and 4B), which is consistent with western blotting results (Figure 4C). In addition, the CSC percentage—and markers such as CD133 and CD44—were significantly reduced after knockdown of circHPS5 in HCC cells (Figures 4D–4F), while overexpression of circHPS5 exerted the opposite effects (Figure S3).

METTL3 facilitates the m6A modification process and circHPS5 expression

Given that specific m6A modification controls the accumulation of a subset of circRNAs,[11] we applied methylated RNA immunoprecipitation (MeRIP) sequencing to detect the m6A mRNA, lncRNA, and circRNA modification profiles of two pairs of HCC tissues and adjacent tissues (Figure S4A). The expression distribution map shows the normalized expression intensity and abundance after the sequence is aligned to the genome (Figure S4B). The sample correlation diagram

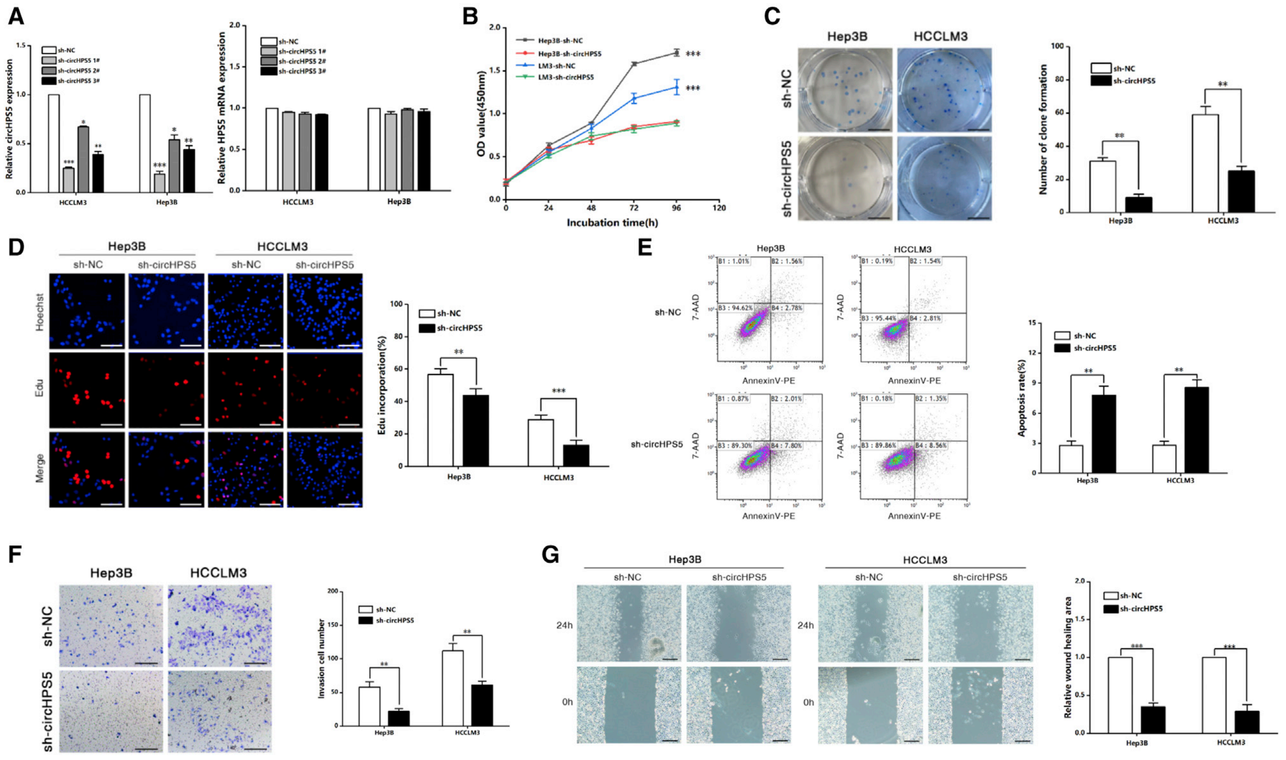

Figure 3. circHPS5 promotes HCC cell proliferation and migration

(A) shRNA against circHPS5 was designed to silence circHPS5 (sh-circHPS5) in HCC cells. Left: The expression of circHPS5 was evaluated using qRT–PCR; Right: The expression of HPS5 mRNA was evaluated using qRT-PCR. (B) The growth curves of cells were constructed using CCK-8 assays after transfection with sh-circHPS5 or sh-NC. (C) A colony formation assay was performed to evaluate cell proliferation. (D) EdU assays of HCC cells transfected with sh-circHPS5 or sh-NC were performed to evaluate cell proliferation. Scale bar,50 μm. (E) Flow cytometry was used to assess cell apoptosis. (F) Transwell experiments were used to assess cell invasion. Scale bar,50 μm. (G) The motility of cells transfected with sh-circHPS5 or sh-NC was examined using wound healing assays. Scale bar,100 μm. $^{**}p < 0.01$, $^{***}p < 0.001$.

verifies that the involved biological experiments can be repeated with little variation in results, ensuring that reliable results can be obtained from subsequent differential gene analysis (Figure S4C). We used the sequencing information of the input library as RNA-seq information to analyze differences in the expression levels of all circRNAs in HCC and noncarcinoma tissues (Figure 5A). We analyzed the association between genes with m6A modification and the RNA expression level of genes and found key genes for follow-up research. To our surprise, circHPS5 was hypermethylated and highly expressed in HCC tissues (Figure 5B). The pie chart shows the distribution of m6A peaks in different genetic environments, mainly in the coding sequence (CDS) region (Figure S4D). We used HOMER software to perform motif analysis of peaks and found that the typical motif modified by m6A is “GGAC” (Figure S4E). GO and KEGG analyses suggested that genes with differential expression and m6A modification were mainly enriched in neoplasms and metabolic channels (Figures 5C and 5D). In particular, we cross-analyzed the circRNA with m6A modification in four tissues and found that the level of the m6A modification in circRNAs was different in different tissues (Figure 5E). The SRAMP prediction website revealed that circHPS5 is highly m6A modified (Figures S4F), and 5F shows the specific structure of the circHPS5 m6A modification, consistent with the MeRIP sequence results. Further m6A RNA-binding protein immunoprecipitation (RIP) experiments confirmed that circHPS5 and m6A antibodies had binding sites (Figure 5G). A previous study reported that the methylase METTL3 directs the biogenesis of a subset of circRNAs;[11] therefore, we explored the association between METTL3 and circHPS5. We found that after knockdown of METTL3 expression via si-METTL3, the expression of circHPS5 decreased significantly, but that of pre-HPS5 and lin-HPS5 increased significantly (Figure 5H). These results indicate that METTL3 can mediate the fate of HPS5 transcripts, mainly to generate circHPS5, rather than processing into HPS5 mRNA.

YTHDC1 facilitates the cytoplasmic output of circHPS5 with m6A modification

Based on the abundant expression of circHPS5 in the cytoplasm and its high methylation level, as well as the system of the m6A modification process and YTHDC1 binding to promote molecular nuclear translocation,[11] we hypothesized that circHPS5 m6A modification sites combined with YTHDC1 can promote circHPS5 nuclear translocation. Next, we performed RNA pulldown experiments to screen circHPS5-interacting proteins. Although common methylases, including METTL3, YTHDC1, YTHDC2, YTHDF1, YTHDF2, and

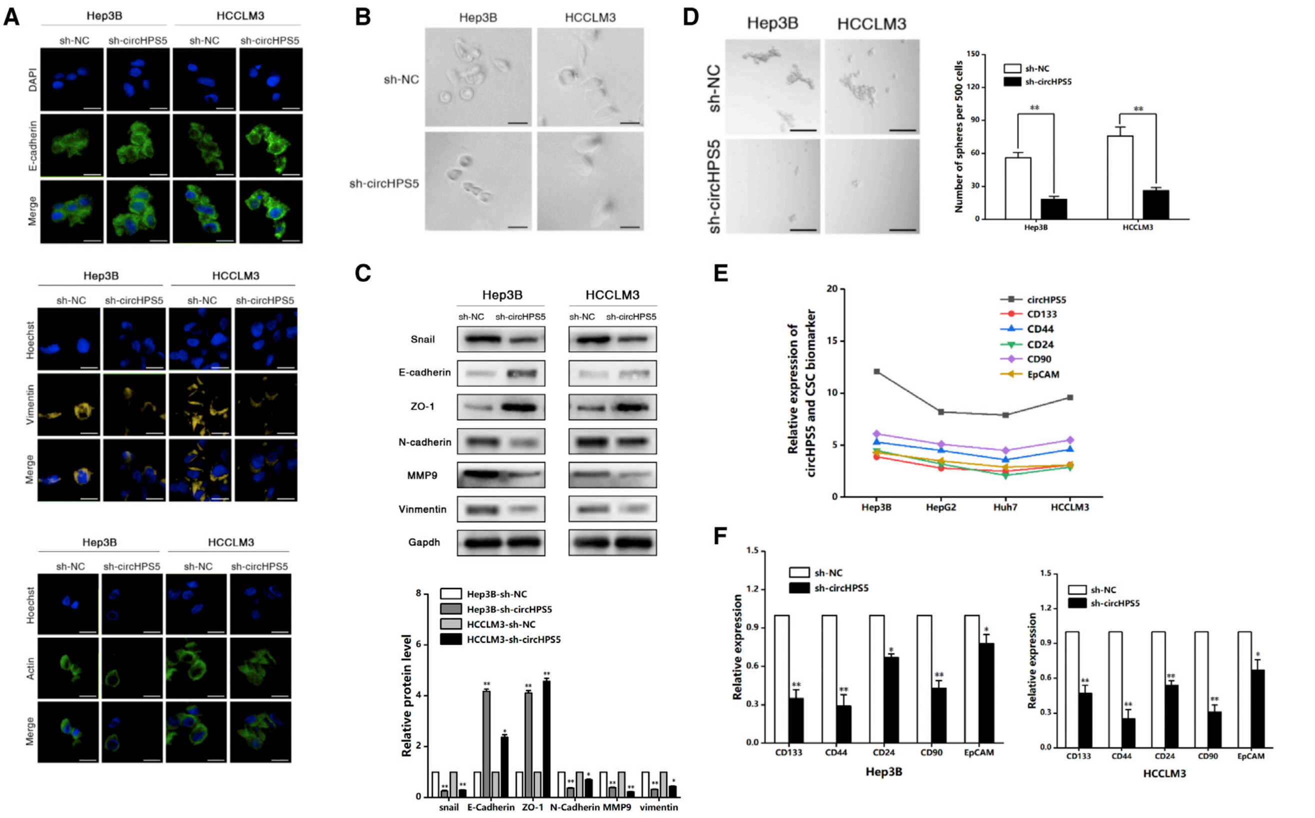

Figure 4. circHPS5 promotes EMT and a CSC phenotype in HCC

(A) Immunofluorescence was employed to detect the expression of E-cadherin, vimentin, and α-actin. Scale bar,10um. (B) Morphological changes associated with EMT in cancer cells. Scale bar,10um. (C) EMT-related protein expression in the sh-NC and sh-circHPS5 groups. (D) Morphological changes associated with CSCs among cancer cells. Scale bar,100um. (E and F) CSC-related marker expression in the sh-NC and sh-circHPS5 groups. *p < 0.05, **p < 0.01.

IGF2BP3, were all positive;YTHDC1 showed the highest expression and thus was used in subsequent studies (Figures 6A and 6B). Further RIP experiments demonstrated enrichment of circHPS5 in complexes precipitated with antibody against YTHDC1 compared to those precipitated with control IgG (Figure 6C). Cytoplasmic and nuclear mRNA fractionation experiments indicated that knockdown of YTHDC1 increased the nuclear circHPS5 content, while the dysregulation of nuclear circHPS5 attributed to si-YTHDC1 was recovered by overexpression of wild-type (WT) but not mutant YTHDC1 (Figure 6D). FISH further confirmed that the increased nuclear staining of circHPS5 caused by si-YTHDC1 was rescued by overexpression of WT but not mutant YTHDC1 (Figure 6E). The above results indicate that YTHDC1 can indeed expedite the cytoplasmic output of m6A-modified circHPS5.

circHPS5 facilitates HCC development by sponging miR-370 to regulate HMGA2

Considering that circRNAs can bind to different miRNAs and regulate downstream genes,[12] we searched two databases (Target Scan, RegRNA) and found that there are three miRNAs (miR-1183, miR-1299, miR-370) in the intersection, which may have binding sites for circHPS5 (Figure 7A). We further tested the expression of these three miRNAs in cells and found that their expression was downregulated in HCC cells compared with normal hepatocytes, with miR-370 showing the greatest decrease (Figure 7B). Pulldown experiments further confirmed the binding of circHPS5 and miR-370 in HCCLM3 and Hep3B cells (Figure 7C). Next, we evaluated the role of miR-370 in HCC cell migration and proliferation. As revealed from the results, miR-370 inhibition induced EMT and CSC phenotypes, promoting HCC cell migration and proliferation, but at the same time, inhibiting circHPS5 expression counteracted this effect (Figures 7D–7I and S5).

By using the CircInteractome Information Base (https://circinteractome.nia.nih.gov/) for bioinformatics analysis, we found that circHPS5 possessed a complementary sequence to the miR-370 seed region. According to miRanda database prediction (http://mirdb.org/), miR-370 can target the HMGA2 mRNA 3′ UTR. TCGA database analysis showed that HMGA2 was upregulated in HCC tissues compared to normal tissues (Figure 8A) and positively linked with TNM stage (Figure 8B). Kaplan–Meier survival curves revealed that patients with higher HMGA2 expression showed worse survival (Figure 8C). To demonstrate the mutual interaction of circHPS5, miR-370 and HMGA2, luciferase reporter experiments were performed, and the results revealed that inhibition of miR-370 significantly enhanced the

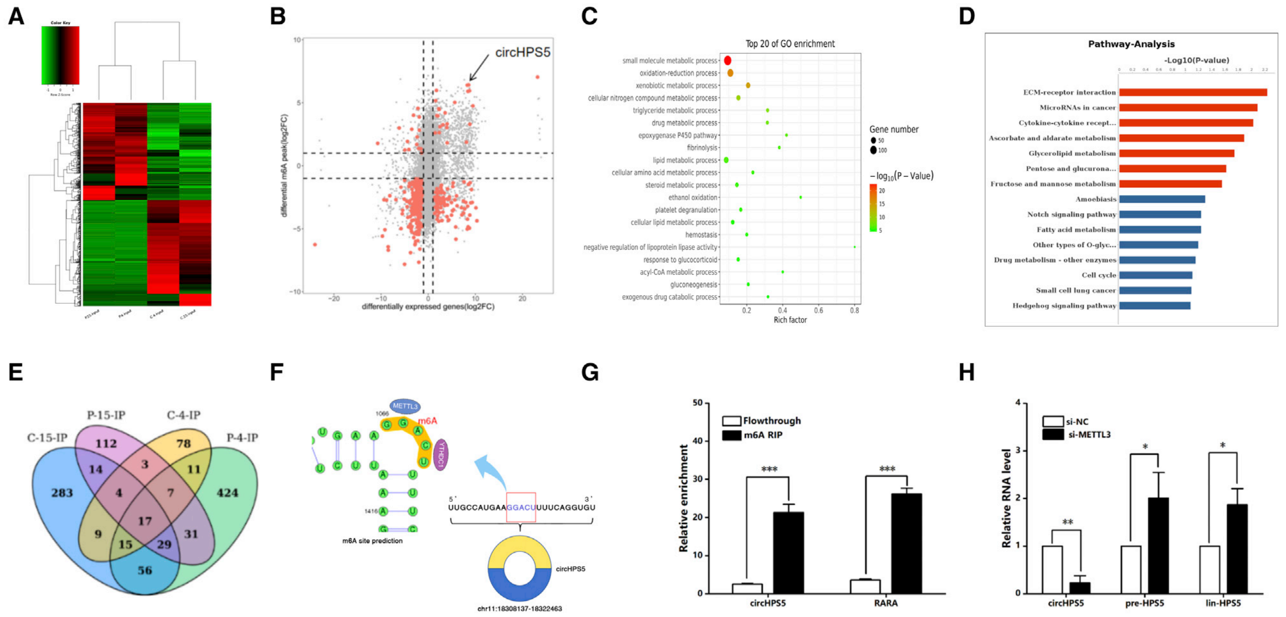

Figure 5. METTL3 promotes m6A modification and expression of circHPS5

(A) m6A levels were detected in two pairs of HCC and adjacent normal tissues via methylated RNA immunoprecipitation (MeRIP) sequencing. "C" represents "cancer," and "P" represents "Normal tissue adjacent to cancer." (B) The association between genes with m6A modification and RNA gene expression levels was analyzed. There were four types of association analysis results: hypermethylated-down (hypermethylated-RNA level down); hypermethylated-up (hypermethylated-RNA level up); hypomethylated-down (demethylated-RNA level down); and hypomethylated-up (demethylated-RNA level upregulated). (C) and (D) GO and KEGG analyses of circRNAs with different m6A modification levels in the HCC group and normal group. (E) The m6A-modified circRNAs in four tissues were cross-analyzed. (F) The SRAMP prediction website revealed the m6A motif structure of circHPS5. (G) MeRIP assays showing the association between circHPS5 and m6A. (H) After knockdown of the methylase METTL3, the expression of circHPS5, pre-HPS5 and lin-HPS5 was detected via qRT–PCR. **p < 0.01, ***p < 0.001.

activity of a circHPS5-WT or HMGA2-WT reporter but not that of a mutant reporter (Figure 8D–8F). In addition, the HMGA2 mutation vector with downregulated circHPS5 and miR-370 expression, further verified the binding sites among circHPS5, miR-370, and HMGA2 (Figure 8G). FISH experiments further confirmed that circHPS5 and miR-370 were colocalized in the cytoplasm (Figure 8H). qRT-PCR confirmed that circHPS5 knockdown decreased the HMGA2 expression level, while inhibition of miR-370 offset this effect (Figure 8I). Western blot analysis further confirmed that inhibition of miR-370 upregulated HMGA2 protein expression, whereas simultaneous sh-circHPS5 treatment counteracted this effect in HCC cells (Figures 8J and 8K), consistent with the HMGA2 mRNA expression results.

circHPS5 facilitates the growth of HCC *in vivo*

To explore the association between circHPS5 and HCC development *in vivo*, HCCLM3 and Hep3B cells transfected with sh-circHPS5, or sh-NC, were injected into nude mice to construct a xenograft neoplasm system (Figure 9A). In addition, sh-circHPS5+methylase inhibitor (SAH) were added to explore the anticarcinoma effect (dimethyl sulfoxide [DMSO] as the control). As revealed by the findings, circHPS5 downregulation effectively decreased the volume and weight of tumors in nude mice, and SAH addition further enhanced this effect (Figure 9B and 9C). The results of hematoxylin-eosin (HE) staining of mouse neoplasms showed that combined sh-circHPS5+SAH treatment significantly reduced the infiltration of carcinoma cells. Moreover, immunohistochemistry (IHC) results revealed that KI67 expression was noticeably reduced (Figure 9D).

DISCUSSION

Here, circHPS5 was demonstrated to exhibit noticeably higher expression in HCC tissues and cells than in normal tissues and cells. Clinicopathological features illustrated that overexpression of circHPS5 indicated a lower total survival period for HCC patients. The relationships between circRNAs and HCC development are being progressively studied clinically. Xiao-Yong Huang et al. reported that circMET (circ_0082002) was overexpressed in HCC neoplasms, and circMET expression displayed a relationship with survival and recurrence in HCC cases.[13] In addition, plasma circRNAs have been verified as diagnostic markers of HCC.[14,15] Here, circHPS5 was verified to be a promising biomarker for HCC diagnosis and prognosis. The acquisition of epithelial-mesenchymal transition and the existence of a subpopulation of cancer stem-like cells are associated with malignant behavior. We found that circHPS5 overexpression can facilitate EMT and CSC phenotypes, further promoting HCC migration and proliferation processes.

In addition, m6A has been shown to be a plentiful transcription-related modification in mRNAs and ncRNAs, including circRNAs. This has broadly been implicated in posttranscription-related mRNA, and influences exerted by m6A modification on cellular circRNA biological

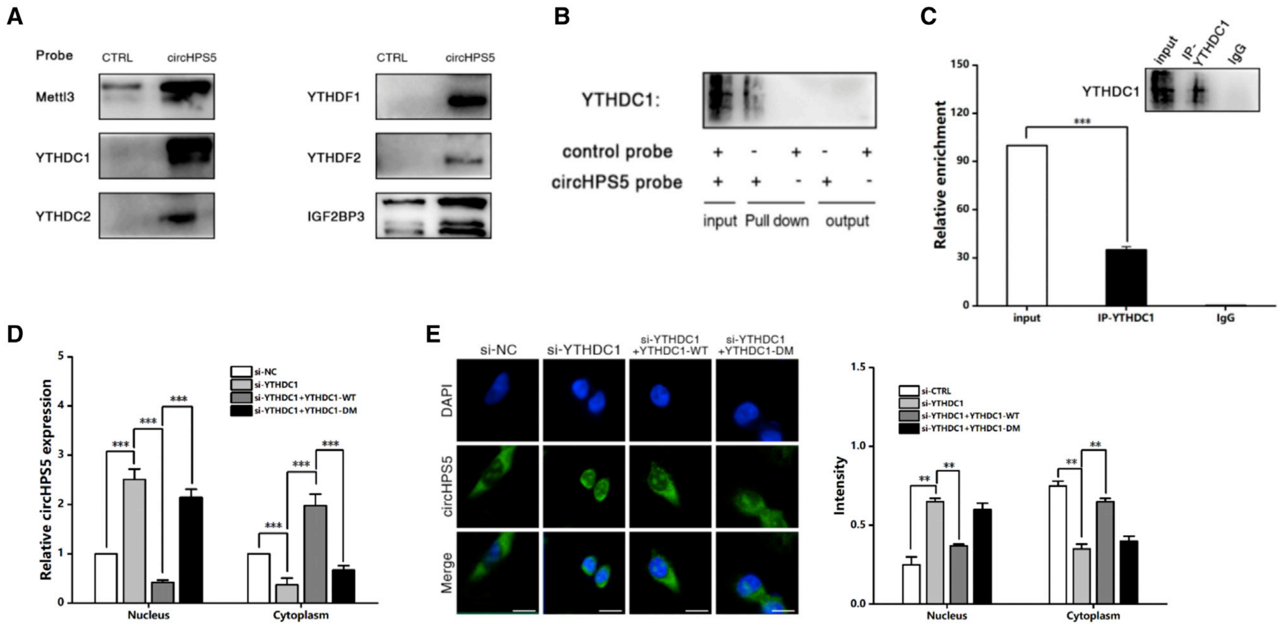

Figure 6. YTHDC1 promotes cytoplasmic export of m6A-modified circHPS5

(A) Immunoblot analysis of YTHDC1 after a pulldown assay showing its specific association with circHPS5. (B) RIP assays showing the association of YTHDC1 with circHPS5. IgG antibody served as a control. (C) YTHDC1 interacted with circHPS5 at the GGACU motif of circHPS5. (D) Cytoplasmic and nuclear mRNA fractionation experiments showing the knockdown of YTHDC1 (WT/mutant) and the expression of circHPS5 in different locations. (E) RNA-FISH showing that the increased nuclear staining of circHPS5 caused by si-YTHDC1 was rescued by overexpression of WT but not mutant YTHDC1. Scale bar,10um. **p < 0.01, ***p < 0.001.

aspects are rarely understood. circRNA biogenesis has been extensively analyzed from broad perspectives. Nevertheless, circRNAs are primarily reported to localize in the cytoplasm.[16,17] For this reason, the systems regulating nuclear-cytoplasmic circRNA output should be explored. According to Chen et al., the m6A methylation process in colorectal carcinoma (CRC) cells mediates the cytoplasmic output of a crucial oncogenic circNSun2. Under m6A modification, circNSun2 in the nucleus can be considered YTHDC1 and transported to the cytoplasm. Next, circNSun2 can stabilize HMGA2 mRNA through circNSun2/insulin-like development element 2 mRNA binding protein 2 (IGF2BP2)/HMGA2 complex formation. Finally, CRC cell invasion and liver metastasis are induced.[18] The YTHDC1 protein has recently attracted attention. Chao Xu et al. reported the crystal structures of the YTHDC1 YTH domain, several YTH domain families, and its complex with an m6A-modified RNA. Relevant structure-related analyses, a transcriptome-broad identification process for YTHDC1 binding sites, and biochemically related experiments revealed the particular m6A-YTH binding mode and explained the preferential m6A sequence recognition process by YTHDC1.[19] In addition, according to Roundtree Ian A et al., the m6A-binding protein YTHDC1 mediates the nucleus-cytoplasm output of methylated mRNA in HeLa cells. YTHDC1 knockdown extends residence time in terms of nuclear mRNA with m6A, with transcripts accumulating in the nucleus and relevant depletion in the cytoplasm. YTHDC1 interacts with the splicing element and SRSF3 (i.e., nuclear output adapting element protein) facilitating RNA binding to NXF1 and SRSF3. Such an effect exerted by YTHDC1 improves the likely utility of the mRNA chemical modification process and supports a novel m6A paradigm as an obvious biochemically related entity to achieve mammalian mRNA metabolism and selective processing.[20] The nucleus-cytoplasm output of circHPS5 relies upon the m6A modification process and is mediated by recruiting YTHDC1, consistent with existing research.[18]

We demonstrated that circHPS5 can act as a miR-370 sponge to regulate HMGA2 expression and further accelerate the tumorigenesis of HCC cells. It has been reported that miR-370 impacts various carcinomas. For instance, Gao Yong-Tao et al. illustrated that miR-370 upregulation recovered the sensitivity of glioblastoma multiforme to temozolomide by impacting MGMT expression.[21] Interestingly, Lulli Valentina et al. identified a vital effect exerted by miR-370 in regulating the glioblastoma development process, indicating that miR-370-3p acts as a neoplasm suppressor element; suppressing glioma cell growth; migration and invasion through HIF1A, HMGA2, and lncRNA NEAT1 targeting; and acting as a potential alternative to treat glioblastoma patients.[22] It has been reported that miR-370-3p reduces ulcerative colitis-associated CRC in mice by suppressing inflammation responses and the epithelial-mesenchymal transition process[23] in HCC. As suggested previously, miR-370 is a neoplasm suppressor via inhibition of the MAPK/JNK signaling pathway by targeting BEX2.[24] The results of the present study further verified that miR-370 inhibits HCC cell migration and proliferation processes. HMGA2 essentially impacts embryogenesis and is an oncoprotein. HMGA2 exhibits high expression in various human carcinomas and is employed as a prognostic marker.[25] Previous studies have confirmed

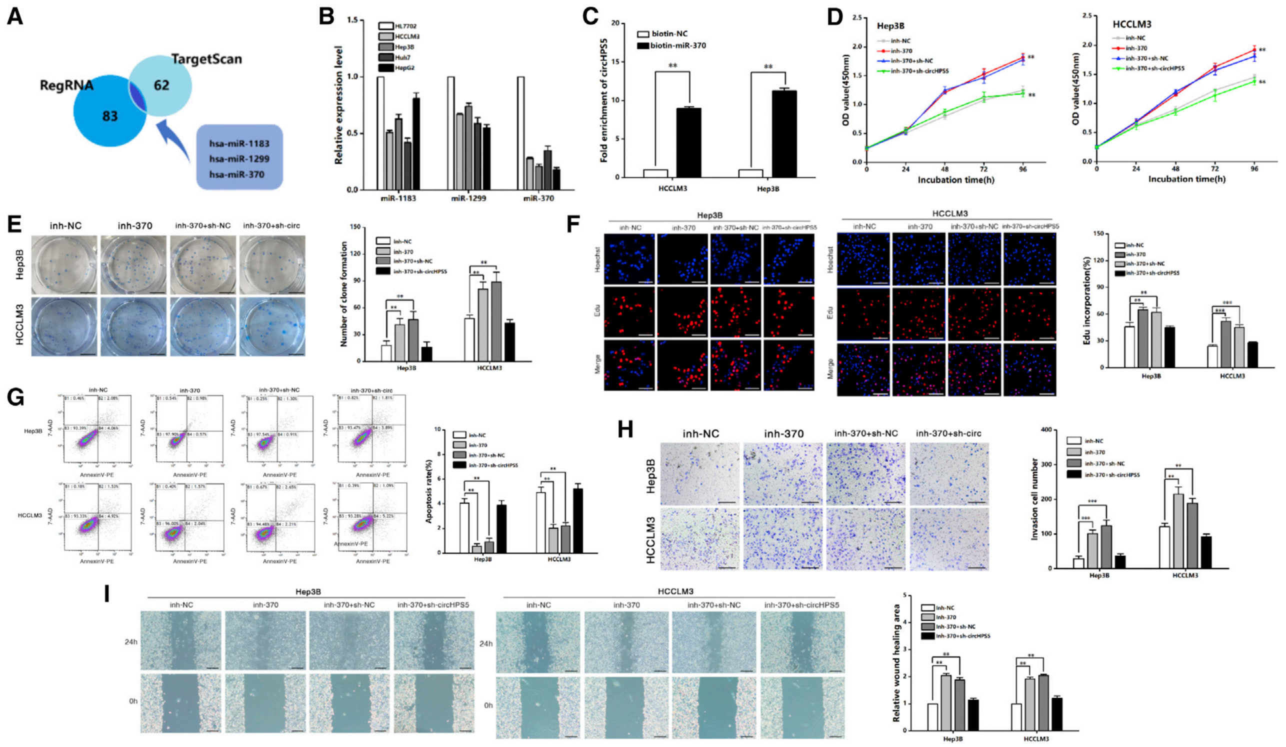

Figure 7. circHPS5 promotes HCC development by sponging miR-370

(A) Two databases (TargetScan and RegRNA) were analyzed, and three miRNAs (miR-1183, miR-1299, and miR-370) that may have binding sites with circHPS5 were identified in the intersection. (B) Expression of the three miRNAs in HCC cells. (C) A pulldown assay further confirmed the binding of circHPS5 and miR-370 in HCC cell lines. (D) Growth curves of cells were constructed using CCK-8 assays after cotransfection with sh-circHPS5 and miR-370 inhibitor. (E) A colony formation assay was performed to evaluate cell proliferation. (F) EdU assays of HCC cells cotransfected with sh-circHPS5 and miR-370 inhibitor were performed to evaluate cell proliferation. Scale bar, 50um. (G) Flow cytometry was used to assess cell apoptosis. (H) Transwell experiments were used to assess cell invasion. Scale bar,50um. (I) Wound healing assay. Scale bar, 100um. $^{**}p < 0.01$, $^{***}p < 0.001$.

that noncoding RNAs promote HCC development by targeting HMGA2.[26,27] In conclusion, we identified a novel circular RNA, termed circHPS5, whose expression was increased in tissues from HCC patients and in HCC cell lines. m6A modification of circHPS5 expedites cytoplasmic output and facilitates EMT and CSC phenotypes, promoting HCC migration and proliferation processes by acting as a miR-370 sponge to regulate HMGA2 expression. Thus, this study reveals a novel potential biomarker and therapeutic target for HCC.

MATERIALS AND METHODS

circRNA microarray

Using four identical arrays on a slide, a CapitalBio Technology Human CircRNA Array v2 was established. A total of three paired carcinoma tissues and paracancerous normal tissues were tested. We employed GeneSpring software V13.0 to study the circRNA array information for quality control, normalizing the process and summarizing the information.

m6A sequencing

This study measured two pairs of HCC carcinoma and paracancerous tissues with m6A-specific antibodies to immunoprecipitate RNA fragments with m6A modification in the cell and subjected the enriched circRNA fragments to high-throughput sequencing. Combined with bioinformatics analysis, the m6A modification process can be systematically studied within the scope of the transcriptome.

Cases and tissue specimen collection

This work was approved by the First Affiliated Hospital of Nanjing Medical University. We performed the informing process in this study in a manner consistent with the Declaration of Helsinki. In advance of the study, the patients provided written informed consent. At the Hepatobiliary/Liver Transplantation Center, human HCC and normal tissues were collected from 46 patients who underwent surgeries under informed consent. All the patients were followed-up regularly, and the total overall survival (OS) period ranged from the date of surgery to the date of death or the last follow-up visit.

Cell cultures

Shanghai Institutes for Biological Sciences, China provided human HCC cell lines and the human liver cell line HL7702. All cell lines were cultured in DMEM (Life Technologies, USA) supplemented with 10% fetal bovine serum (GIBCO, Australia), 100 IU/ml penicillin

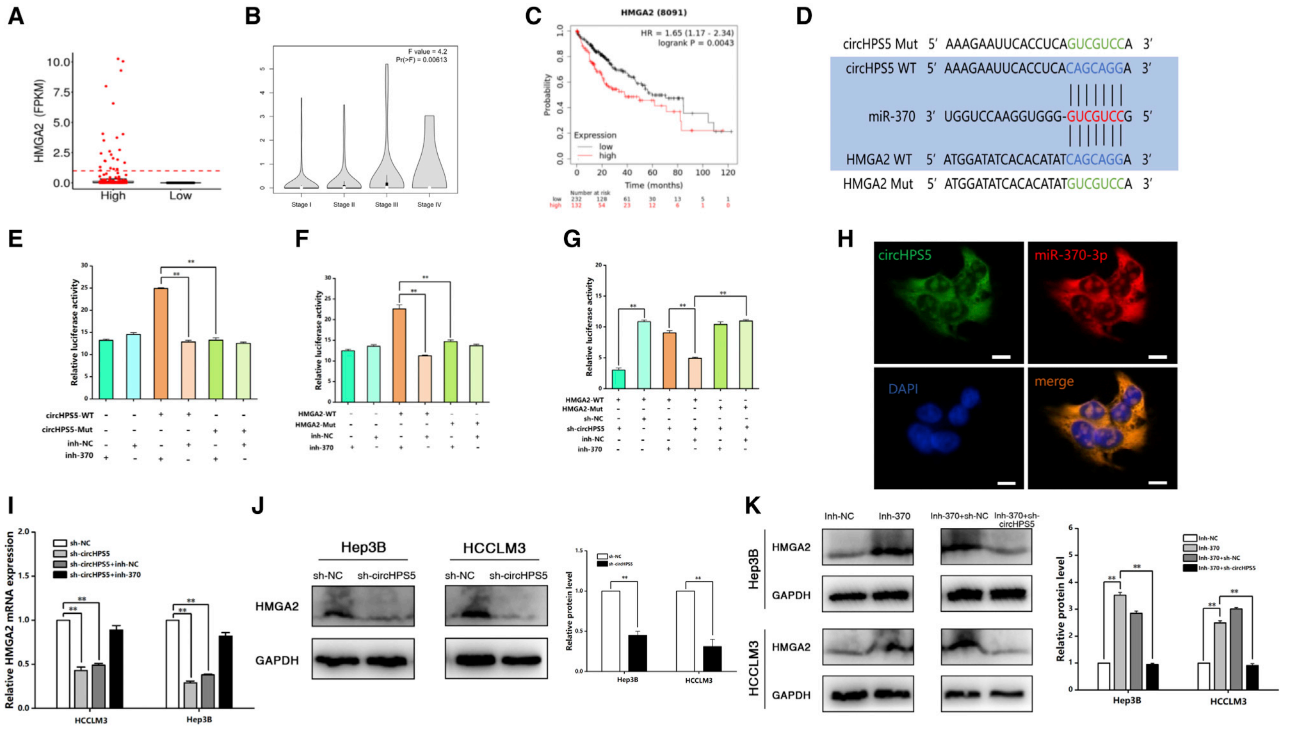

Figure 8. circHPS5 promotes HCC progression by sponging miR-370 to regulate HMGA2

(A) The expression of HMGA2 in HCC tissues and normal tissues via TCGA database prediction. (B) The association between HMGA2 expression and TNM stage. (C) Kaplan–Meier survival curve showing the relationship between HMGA2 and overall survival time. (D–G) circHPS5 possesses a complementary sequence to the miR-370 seed region according to bioinformatics analysis. Relative luciferase activities were analyzed in 293T cells cotransfected with miR-370 inhibitor and the luciferase reporter vectors pGL3-circHPS5-WT or pGL3-circHPS5-Mut. Relative luciferase activities were analyzed in 293T cells cotransfected with miR-370 inhibitor and the luciferase reporter vectors pGL3-HMGA2-WT or pGL3-HMGA2-Mut. In addition, relative luciferase activities were analyzed in 293T cells cotransfected with miR-370 inhibitor or sh-circHPS5 and the luciferase reporter vectors pGL3-HMGA2-WT or pGL3-HMGA2-Mut. (H) FISH assay showing the location of circHPS5 and miR-370. Scale bar, 10um. (I) The relative expression of HMGA2 mRNA in cells transfected with sh-circHPS5 and miR-370 inhibitor was evaluated using qRT-qPCR. (J and K) The relative expression of HMGA2 protein in cells transfected with sh-circHPS5 and/or miR-370 inhibitor was evaluated using western blotting. **p < 0.01.

and 100 mg/ml streptomycin in a humidified incubator with 5% CO_2 at 37°C.

Sanger sequencing

For determination of their full length, this study inserted the amplification products into a T-vector for Sanger sequencing. Divergent primers were designed to confirm the back-splice junction of circHPS5. Sanger sequencing was performed by Realgene (China).

Quantitative reverse transcription polymerase reaction (qRT-PCR)

Using the manufacturer's protocol, total RNA was isolated from tissues and cells with TRIzol reagent (Invitrogen, USA). Based on a reverse transcription kit (Takara, Japan), cDNA was synthesized for circRNA and mRNA; based on a RiboBio reverse transcription kit (Guangzhou, China), overall RNA was reverse transcribed for miRNA. Using a SYBR Green PCR Kit (Takara, Japan), we quantified mRNA and circular RNA; using a PCR Kit (RiboBio, China), miRNA PCR was carried out. All primer sequences are listed in Table S2. GAPDH was used to normalize the mRNA and circRNA expression levels, and U6 was used to normalize the miRNA expression levels before calculation.

Nucleus-cytoplasm fractionation

First, 1×10^6 HCC cells were washed two times with PBS. The cell layer was scraped in 500 μL of PBS and centrifuged for 5 min at $500 \times g$ at 4°C. Using a PARIS KIT 50 RXNS (Life AM1921, USA), nuclear and cytoplasmic RNA from cultured HCC cells was isolated according to the manufacturer's instructions. We performed qRT-PCR for circHPS5 abundance detection.

Oligonucleotide transfection and stable transfection

sh-circHPS5, miR-370 inhibiting elements and their relevant regulating oligonucleotides were designed and synthesized by RiboBio (China). All transfections were conducted with miRNA inhibitor and sh-circHPS5. Lipofectamine 2000 reagent (Invitrogen, USA) was added to the transfection medium. In compliance with the manufacturer's instructions, human HCC cell lines were infected with lentivirus at a multiplicity of infection of 50. All cell lines underwent

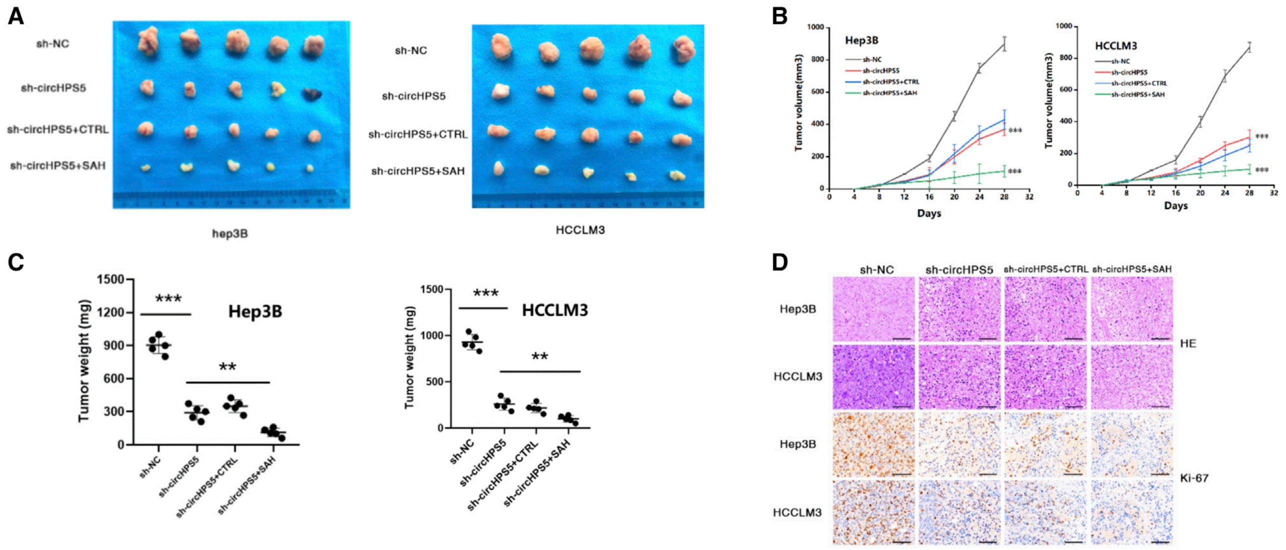

Figure 9. circHPS5 promotes HCC growth *in vivo*

(A) Representative images of HCC tumor-bearing BALB/c nude mice and xenograft HCC tumors. (B) Growth curves of xenograft tumors. The tumor volumes were measured every 4 days. (C) The relative weights of tumors were evaluated. (D) Immunohistochemical analysis showing KI67 staining in tumors formed by HCC cells. Scale bar, 50um. $^{**}p < 0.01$, $^{***}p < 0.001$.

the selection process with 2 μg/mL puromycin for two weeks. All probe sequences are listed in Table S2.

Fluorescence *in situ* hybridization (FISH)

A fluorescence *in situ* hybridization experiment was conducted for circHPS5 and miR-370 detection using a Fluorescence *in Situ* Hybridization Kit (RiboBio, China). circHPS5 was captured with a Cy5-labeled probe; miR-370 was captured with a Cy3-labeled probe. After the prehybridization process, the circHPS5 and miR-370 probes were hybridized in prepared hybridization buffer with HCC cells. Nuclei were stained with 4,6-diamidino-2-phenylindole (DAPI). Confocal microscopy was adopted to more effectively visualize the presence of circHPS5 and miR-370. All probe sequences are listed in Table S2.

Cell proliferation experiments

In the clone forming experiments, HCC cells under transfection received the seeding inside 6-well plates at 1000 cells per well density. After 10 days, the cells received the fixing based on the use of methanol, followed by the staining with GIMSA. Eventually, colonies received the imaging and counting. For CCK8 assay, HCC cells were seeded in 96-well plates at 4000 cells per well. Seeded cells received 10 μl of CCK-8 solution (RiboBio, China) at 0 h, 24 h, 48 h, 72 h, and 96 h. Subsequently, cell absorbance at 450 nm was analysed at the respective times with a microplate reader in accordance with the manufacturer's instructions (Synergy4, USA). Using a Cell-Light EdU DNA Cell Proliferation Kit (RiboBio, China), an EdU experiment was performed to assess the proliferation of cells. HCC cells were plated in 24-well plates and cultured for 24 h. The mentioned cell lines were fixed with 4% paraformaldehyde after incubation with 50 mM EdU solution for 2 h. Next, in accordance with the manufacturer's protocol, cell lines underwent a sealing process with Apollo Dye Solution and Hoechst separately. Under an Olympus FSX100 microscope (Olympus, Japan), the EdU cell lines were imaged and counted.

Cell apoptosis assays

HCC cells were stained with AnnexinV-PE and 7-AAD using an apoptosis detection kit (BD Biosciences, USA). FACScan (BD Biosciences, USA) was used to analyze stained cells, and all apoptosis data of different cell lines were analyzed using FlowJo V10 software (Tree Star, USA).

Transwell invasion assays

For this assay, according to the manufacturer's protocol, HCC cells were seeded in the upper chambers with 200 μL of serum-free medium. The Transwell chamber (Corning, USA) was coated with Matrigel mix (BD Biosciences, USA) for the invasion assay. The bottom chamber was filled with medium and 10% FBS as a cancer cell chemoattractant. After incubation for 24 h, cells in the upper chambers were fixed and stained with crystal violet for 15 minutes. For visualization, the cells were photographed and counted in different fields.

Scratch wound experiment

When cell confluence reached approximately 90% at 24 h posttransfection, wounds were created with a 200 μl pipette tip, and the cells were rinsed with medium to remove free-floating cells and debris. The medium was added, and the culture plates were incubated at 37°C. Wound healing was assessed at various time points. Furthermore, representative scrape lines were imaged.

Sphere formation assay

Cells were seeded into six-well ultralow attachment plates (Corning, USA) in DMEM/F12 (GIBCO, USA) supplemented with 2% B27, 10 μng/ml EGF and 10 μng/ml basic FGF (GIBCO, USA). After 7 days of culture, HCC cell pellets were grown, centrifuged, and digested with StemPro Accutase Cell Dissociation Reagent (Invitrogen, USA) and then seeded (500 cells/well) to form another secondary pellet. Spheres with a diameter > 75 μm were counted.

RNA-binding protein immunoprecipitation (RIP)

HCC cells were harvested 48 h after the transfection process and lysed in RIP lysis buffer on ice for 30 min. After centrifugation, the supernatant was incubated with antibodies and 30 μl of Protein-A/G agarose beads (Roche, USA). After incubation overnight, the immune complexes were centrifuged and subsequently cleaned six times using cleaning buffer. Immunoprecipitated RNA was analyzed via qRT-PCR.

Biotin-coupled probe RNA pulldown experiment

In compliance with the manufacturer's instructions, cell lysate was incubated with magnetic beads coated with streptavidin (Invitrogen, USA) to pull biotin-conjugated RNA complexes down. Through qRT-PCR analysis, the enrichment of circHPS5 in the capture region was assessed. The bound protein was eluted from the packaged beads and analyzed via SDS-PAGE.

Luciferase reporter experiment

Mutant and wild-type sequences of the 3′-UTR of circHPS5 or HMGA2 displaying associations with the miR-370 binding site were designed, synthesized, and inserted into pGL3-basic vectors (Realgene, China). Next, 293T cells were cotransfected with pGL3-basic vectors and miR-370 inhibiting element or sh-circHPS5 lentivirus. After 48 h, the luciferase activity in cotransfected cells was collected and detected in a dual-luciferase reporter experiment (Promega, USA).

Western blotting

Cells were lysed in RIPA lysis buffer. The protein was prepared and quantified using a bicinchoninic acid (BCA) analysis (Beyotime, China). Identical amounts of proteins were separated in 10% SDS–PAGE gels and transferred to PVDF membranes (Millipore, Germany). The proteins were blocked using 5% skim milk powder and incubated with the primary antibodies anti-GAPDH (ab9485) and anti-HMGA2 (ab207301) at 4°C for 12 h. Subsequently, the membranes were incubated with secondary antibody for 2 h. Finally, the blots were detected with an enhanced chemiluminescence kit (Pierce, USA), and the relevant information was obtained using Image Lab Software.

Xenografts in mice

The animal management committee of Nanjing Medical University approved the animal experiments, and all experimentally related processes and animal care were in accordance with the institutional ethics directions for animal-related experimental processes. To create the xenograft neoplasm system, 40 male BALB/c nude mice aged 5 weeks were randomly separated into sh-NC, sh-circHPS5, sh-circHPS5+CTRL, and sh-circHPS5+SAH groups (n = 5 for each group). HCC cells were subcutaneously injected into the axilla of the nude mice. The volume of tumors in all the injected nude mice was determined every 4 days using digital callipers.

Immunohistochemistry (IHC)

In brief, sections embedded in paraffin underwent deparaffinization and rehydration. Peroxidase activity was blocked with 3% hydrogen peroxide. Sections were incubated overnight with a primary antibody against KI67 (ab15580) at 4°C. Next, a biotinylated secondary antibody was incubated with tissue sections, and the sections were then incubated with streptavidin-horseradish peroxidase complex. Diaminobenzidine was used to show immunoreactivity. The sections were counterstained with hematoxylin. The tissue sections were imaged under a fluorescence microscope. KI67 quantitation was performed using Image-Pro Plus 6.0 software according to the integrated optical density/area of the color value.

Statistical analysis

SPSS 19.0 software (IBM, USA) was used for data analysis, and a p value < 0.05 was considered to indicate statistical significance. Comparison of continuous information was based on an individual t test between the two groups, while categorically related data were analyzed with a chi-square test. The Kaplan-Meier approach and a log rank test were the primary applications used to assess survival rates.

Ethics approval and consent to participate

This study was approved by the Institutional Ethics Committee of The First Affiliated Hospital of Nanjing Medical University. Informed consent was obtained from all patients prior to analysis. All animal experimental methods involved in this study are in line with the Declaration of Helsinki and approved by Nanjing Medical University.

Availability of data and material

Data of high-throughput sequencing are deposited publically in GEO: GSE166678.

SUPPLEMENTAL INFORMATION

Supplemental information can be found online at https://doi.org/10.1016/j.omtn.2021.09.001.

ACKNOWLEDGMENTS

We are grateful for the grants from the National Natural Science Key Foundation of China (Grant No.31930020) and National Natural Science Foundation of China (Grant No.81771716).

AUTHOR CONTRIBUTIONS

X.W., W.T., Y.X and D.R. conceived and designed the experiments; D.R.,F.W., C.L., and G.S. performed the experiments; D.R. and F.W. analyzed the data; X.S., X.C., Y.D., W.Z., X.H., Y.X. and J.Z. collected the clinical samples and analyzed the clinical data; F.W. and C.L. performed the bioinformatics and statistical analyses; W.T. and Y.X.

obtained funds; and D.W., Y.X., and W.T. drafted and revised the manuscript. All authors gave final approval of the manuscript.

DECLARATION OF INTERESTS

The authors declare no competing interests.

REFERENCES

1. Siegel, R.L., Miller, K.D., and Jemal, A. (2019). Cancer statistics, 2019. CA Cancer J. Clin. *69*, 7–34.
2. Forner, A., Llovet, J.M., and Bruix, J. (2012). Hepatocellular carcinoma. Lancet *379*, 1245–1255.
3. El-Serag, H.B., and Rudolph, K.L. (2007). Hepatocellular carcinoma: epidemiology and molecular carcinogenesis. Gastroenterology *132*, 2557–2576.
4. Yang, J.D., Hainaut, P., Gores, G.J., Amadou, A., Plymoth, A., and Roberts, L.R. (2019). A global view of hepatocellular carcinoma: trends, risk, prevention and management. Nat. Rev. Gastroenterol. Hepatol. *16*, 589–604.
5. Desrosiers, R., Friderici, K., and Rottman, F. (1974). Identification of methylated nucleosides in messenger RNA from Novikoff hepatoma cells. Proc. Natl. Acad. Sci. USA *71*, 3971–3975.
6. Pan, Y., Ma, P., Liu, Y., Li, W., and Shu, Y. (2018). Multiple functions of m^6A RNA methylation in cancer. J. Hematol. Oncol. *11*, 48.
7. Chen, Y., Lin, Y., Shu, Y., He, J., and Gao, W. (2020). Interaction between N^6-methyladenosine (m^6A) modification and noncoding RNAs in cancer. Mol. Cancer *19*, 94.
8. Capel, B., Swain, A., Nicolis, S., Hacker, A., Walter, M., Koopman, P., Goodfellow, P., and Lovell-Badge, R. (1993). Circular transcripts of the testis-determining gene Sry in adult mouse testis. Cell *73*, 1019–1030.
9. Nigro, J.M., Cho, K.R., Fearon, E.R., Kern, S.E., Ruppert, J.M., Oliner, J.D., Kinzler, K.W., and Vogelstein, B. (1991). Scrambled exons. Cell *64*, 607–613.
10. Yang, Y., Fan, X., Mao, M., Song, X., Wu, P., Zhang, Y., Jin, Y., Yang, Y., Chen, L.L., Wang, Y., et al. (2017). Extensive translation of circular RNAs driven by N^6-methyladenosine. Cell Res. *27*, 626–641.
11. Di Timoteo, G., Dattilo, D., Centrón-Broco, A., Colantoni, A., Guarnacci, M., Rossi, F., Incarnato, D., Oliviero, S., Fatica, A., Morlando, M., and Bozzoni, I. (2020). Modulation of circRNA Metabolism by m^6A Modification. Cell Rep. *31*, 107641.
12. Hansen, T.B., Jensen, T.I., Clausen, B.H., Bramsen, J.B., Finsen, B., Damgaard, C.K., and Kjems, J. (2013). Natural RNA circles function as efficient microRNA sponges. Nature *495*, 384–388.
13. Huang, X.Y., Zhang, P.F., Wei, C.Y., Peng, R., Lu, J.C., Gao, C., Cai, J.B., Yang, X., Fan, J., Ke, A.W., et al. (2020). Circular RNA circMET drives immunosuppression and anti-PD1 therapy resistance in hepatocellular carcinoma via the miR-30-5p/snail/DPP4 axis. Mol. Cancer *19*, 92.
14. Li, Z., Zhou, Y., Yang, G., He, S., Qiu, X., Zhang, L., Deng, Q., and Zheng, F. (2019). Using circular RNA SMARCA5 as a potential novel biomarker for hepatocellular carcinoma. Clin. Chim. Acta *492*, 37–44.
15. Guo, X., Lv, X., Lv, X., Ma, Y., Chen, L., and Chen, Y. (2017). Circulating miR-21 serves as a serum biomarker for hepatocellular carcinoma and correlated with distant metastasis. Oncotarget *8*, 44050–44058.
16. Fang, J., Hong, H., Xue, X., Zhu, X., Jiang, L., Qin, M., Liang, H., and Gao, L. (2019). A novel circular RNA, circFAT1(e2), inhibits gastric cancer progression by targeting miR-548g in the cytoplasm and interacting with YBX1 in the nucleus. Cancer Lett. *442*, 222–232.
17. Tan, S., Sun, D., Pu, W., Gou, Q., Guo, C., Gong, Y., Li, J., Wei, Y.Q., Liu, L., Zhao, Y., and Peng, Y. (2018). Circular RNA F-circEA-2a derived from EML4-ALK fusion gene promotes cell migration and invasion in non-small cell lung cancer. Mol. Cancer *17*, 138.
18. Chen, R.X., Chen, X., Xia, L.P., Zhang, J.X., Pan, Z.Z., Ma, X.D., Han, K., Chen, J.W., Judde, J.G., Deas, O., et al. (2019). N^6-methyladenosine modification of circNSUN2 facilitates cytoplasmic export and stabilizes HMGA2 to promote colorectal liver metastasis. Nat. Commun. *10*, 4695.
19. Xu, C., Wang, X., Liu, K., Roundtree, I.A., Tempel, W., Li, Y., Lu, Z., He, C., and Min, J. (2014). Structural basis for selective binding of m6A RNA by the YTHDC1 YTH domain. Nat. Chem. Biol. *10*, 927–929.
20. Roundtree, I.A., Luo, G.Z., Zhang, Z., Wang, X., Zhou, T., Cui, Y., et al. (2017). YTHDC1 mediates nuclear export of N^6-methyladenosine methylated mRNAs. eLife *6*, e31311.
21. Gao, Y.T., Chen, X.B., and Liu, H.L. (2016). Up-regulation of miR-370-3p restores glioblastoma multiforme sensitivity to temozolomide by influencing MGMT expression. Sci. Rep. *6*, 32972.
22. Lulli, V., Buccarelli, M., Ilari, R., Castellani, G., De Dominicis, C., Di Giamberardino, A., et al. (2020). Mir-370-3p Impairs Glioblastoma Stem-Like Cell Malignancy Regulating a Complex Interplay between HMGA2/HIF1A and the Oncogenic Long Non-Coding RNA (lncRNA) NEAT1. Int. J. Mol. Sci. *21*, 3610.
23. Lin, L., Wang, D., Qu, S., Zhao, H., and Lin, Y. (2020). miR-370-3p Alleviates Ulcerative Colitis-Related Colorectal Cancer in Mice Through Inhibiting the Inflammatory Response and Epithelial-Mesenchymal Transition. Drug Des. Devel. Ther. *14*, 1127–1141.
24. Wang, X., Zhu, W., Xu, C., Wang, F., Zhu, X., Sun, Y., Guo, Y., Fu, X., Zhang, Y., and Zang, Y. (2019). MicroRNA-370 functions as a tumor suppressor in hepatocellular carcinoma via inhibition of the MAPK/JNK signaling pathway by targeting BEX2. J. Hum. Genet. *64*, 1203–1217.
25. Zhang, S., Mo, Q., and Wang, X. (2019). Oncological role of HMGA2 (Review). Int. J. Oncol. *55*, 775–788.
26. Mo, J., Li, B., Zhou, Y., Xu, Y., Jiang, H., Cheng, X., Wu, X., and Zhang, Y. (2019). LINC00473 promotes hepatocellular carcinoma progression via acting as a ceRNA for microRNA-195 and increasing HMGA2 expression. Biomed. Pharmacother. *120*, 109403.
27. Huang, W., Li, J., Guo, X., Zhao, Y., and Yuan, X. (2016). miR-663a inhibits hepatocellular carcinoma cell proliferation and invasion by targeting HMGA2. Biomed. Pharmacother. *81*, 431–438.

OMTN, Volume *26*

Supplemental information

m6A modification of circHPS5 and hepatocellular carcinoma progression through HMGA2 expression

Dawei Rong, Fan Wu, Chen Lu, Guangshun Sun, Xiaoli Shi, Xiaoyuan Chen, Yongjiu Dai, Weizhe Zhong, Xiaopei Hao, Jinren Zhou, Yongxiang Xia, Weiwei Tang, and Xuehao Wang

Supplementary Table 1 Analysis between values and expression level of circHPS5 in HCC

Parameters	Low<br>N=13	High<br>N=33	P-value
**Age(years)**			0.463
＜ 60	8	18	
≥ 60	5	15	
**Gender**			0.305
Male	10	29	
Female	3	4	
**Size(cm)**			0.000*
＜ 5	13	8	
≥ 5	0	25	
**TNM stage**			0.006*
I	12	16	
II–IV	1	17	
**Microvascular invasion**			0.015*
Negative	12	18	
Positive	1	15	
**Virus infection**			0.330
Negative	2	9	
Positive	11	24	

* means P＜0.05

Supplementary Table 2 sequence of all genes and probes

name	sequence
circHPS5-F	TCTGCAACCAGAGTCTTTAAGGT
circHPS5-R	GGTTCAGTCGTGTGTTACGA
HPS5-F	TCGATCCTTCTTGTGTGACACT
HPS5-R	GGTGGCAACGAGAGGAGTTTC
GAPDH-F	GGGAAACTGTGGCGTGAT
GAPDH-R	GAGTGGGTGTCGCTGTTGA
RARA-F	GAAGTGCTTTGAAGTGGGCA
RARA-R	GTCCCAGAGGTCAATGTCCA
miR-1183-F	ACTGACCACTGTAGGTGATGGT
miR-1183-R	GCGAGCACAGAATTAATACGACTCACTATAGG
miR-1299-F	ACACTCCAGCTGGGTTCTGGAAUUCTC
miR-1299-R	CAGTGCGTGTCGTGGAGT
miR-370-3p-RT	GTCGTATCCAGTGCAGGGTCCGAGGTATTCGCACTGGATACGACACCAGG
miR-370-3p-F	GCCTGCTGGGGTGGAACC
miR-370-3p-R	CAGTGCAGGGTCCGAGGT
U6-F	GCTTCGGCAGCACATATACTAAAA
U6-R	CGCTTCACGAATTTGCGTGTCAT
sh-circHPS5-1#	AAGGTTATCCCAGTGAGCATT
sh-circHPS5-2#	GAAGGTTATCCCAGTGAGCAT
sh-circHPS5-3#	GGTTATCCCAGTGAGCATTGT
si-METTL3	GCACAUCCUACUCUUGUAATT
si-YTHDC1	GCAAGGAGUGUUAUCUUAATT

control-probe	UUGUACUACACAAAAGUACUG
circHPS5-probe	ACACAATGCTCACTGGGATAACCTTCATC
miR-370-probe	ACCAGGTTCCACCCCAGCAGGC

Figure S1 Agarose gel electrophoresis was employed for the verification of circHPS5 specificity.

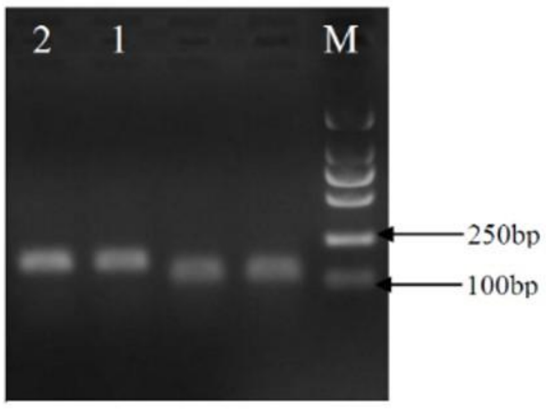

Figure S2 (a) Over-expression of circHPS5 was designed to up-regulate circHPS5 level in HCC cells. (b)The growth curves of cells were measured after transfection with vector and circHPS5 by using CCK-8 assays. (c)Cloning formation assay was performed to evaluate cell proliferation.(d)EdU assays of HCC cells transfected with vector and circHPS5 were performed to evaluate cell proliferation. (e) Flow cytometry was used to assess cell apoptosis.(f)Transwell experiment was used to assess cell invasion.(g) Cell motility was examined in cells transfected with vector or circHPS5 by by wound healing assay. *p < 0.05,**p < 0.01,***p < 0.001.

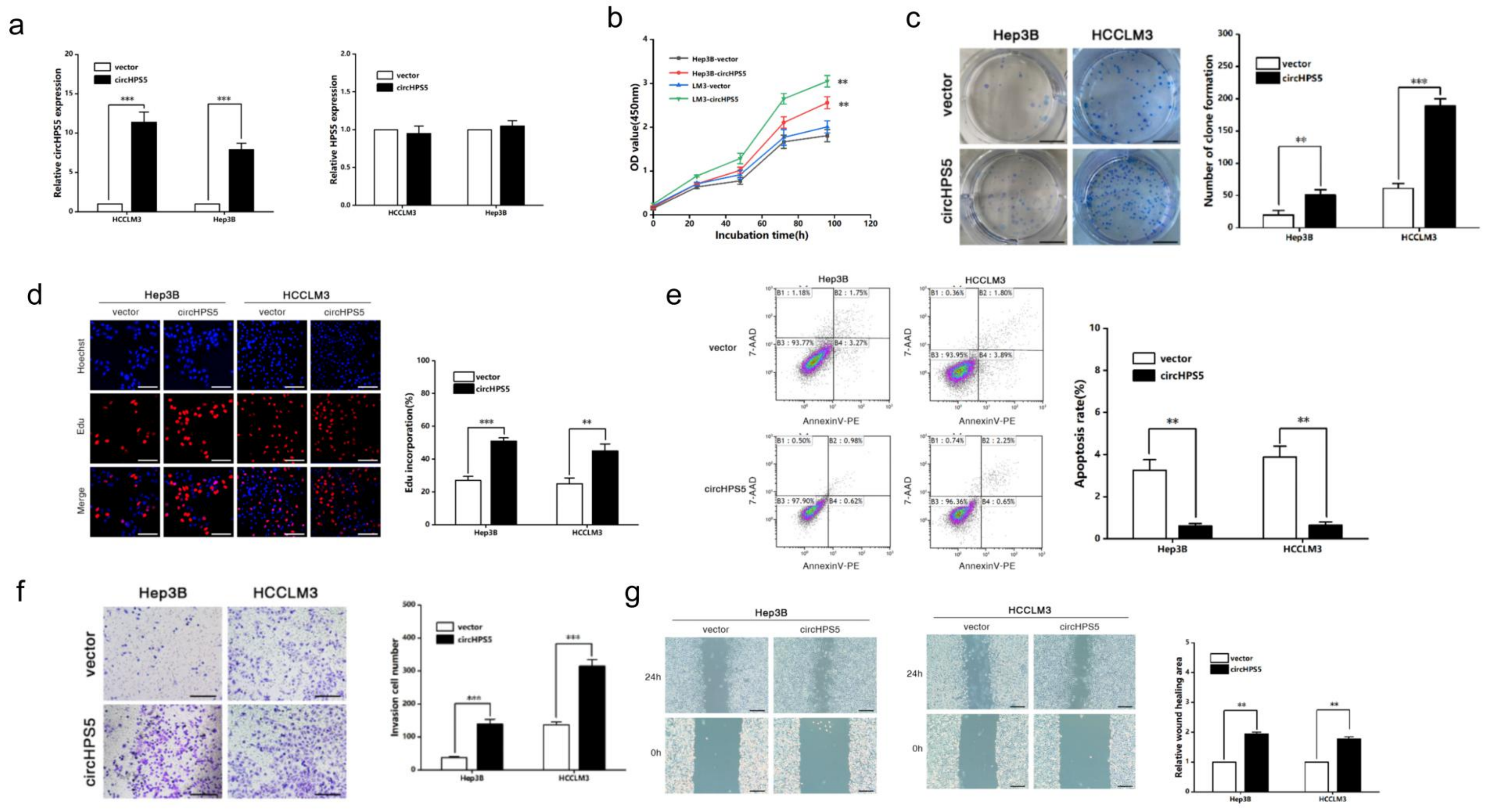

Figure S3(a)Immunofluorescence was employed to detect the expression of E-cadherin, Vimentin ,and α-actin . (b)Morphological changes of EMT in cancer cells. (c)EMT related protein expression in ector and circHPS5 group.(d) Morphological changes of CSC in cancer cells.(e) CSC related markers expression in vector or circHPS5 group. *p < 0.05,**p < 0.01.

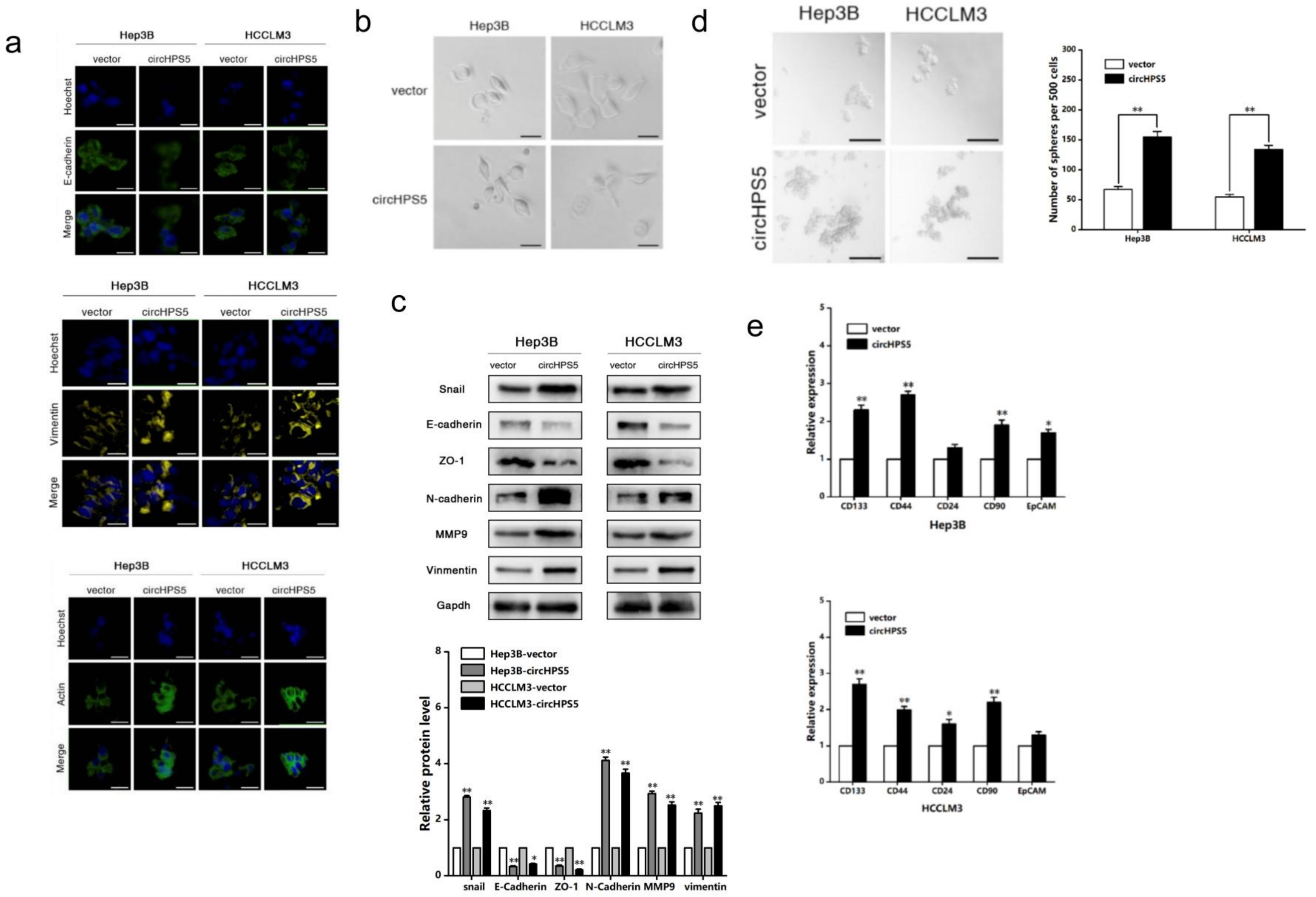

Figure S4(a) Flow chart of methylation sequencing.(b)The expression distribution map describes the normalized expression distribution intensity and abundance after the sequence is aligned to the genome.(c)The sample correlation diagram proves that the involved biological experiment operations can be repeated with little variation, ensuring that the subsequent differential gene analysis can get more reliable results.(d)Peak distribution pie chart on RNA structure.(e)We used HOMER software to perform motif analysis on peaks and found that the typical motif modified by m6A is "GGAC".(f)SRAMP prediction website revealed that circHPS5 was highly m6A modified.

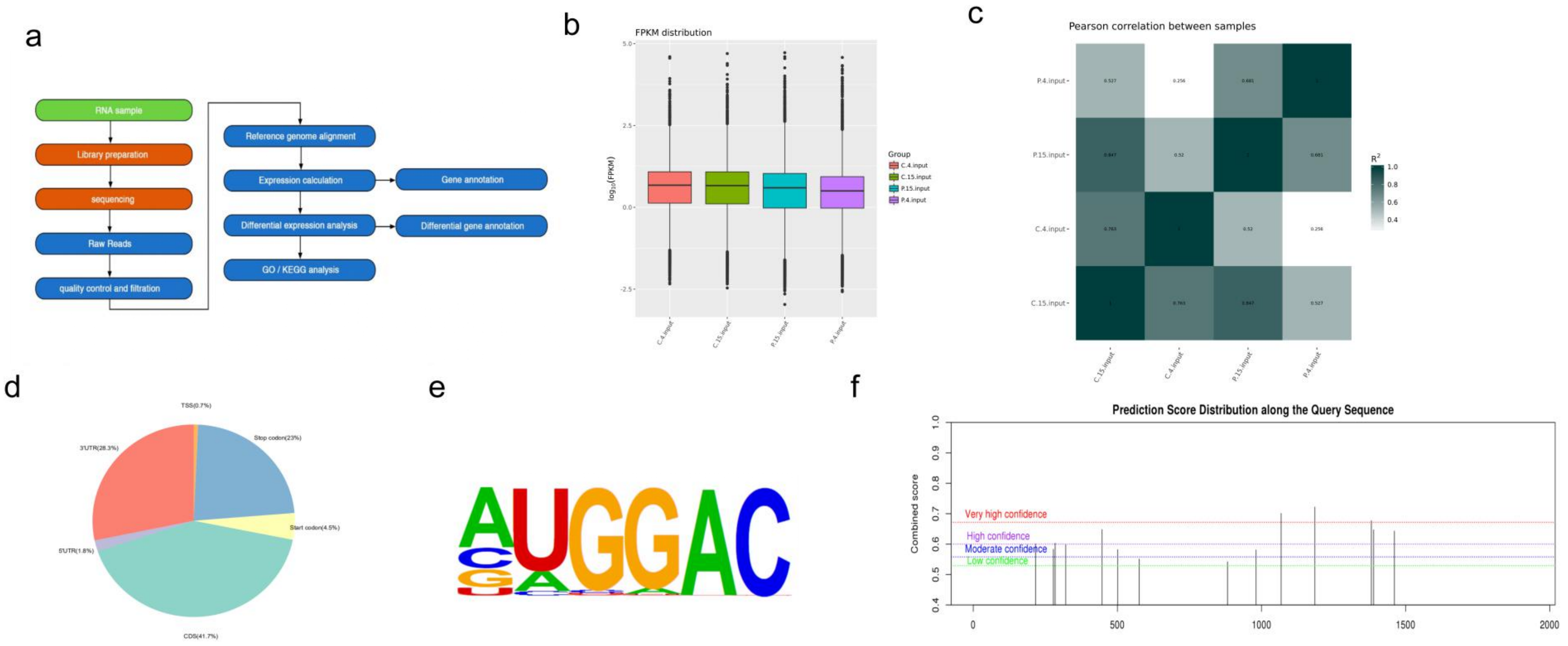

Figure S5 (a) Morphological changes of EMT in cancer cells in different groups. (b)Immunofluorescence was employed to detect the expression of E-cadherin, Vimentin ,and α-actin in different groups.(c)Morphological changes of CSC in cancer cells.(d) CSC related markers expression in different groups. *p < 0.05,**p < 0.01,***p < 0.001.

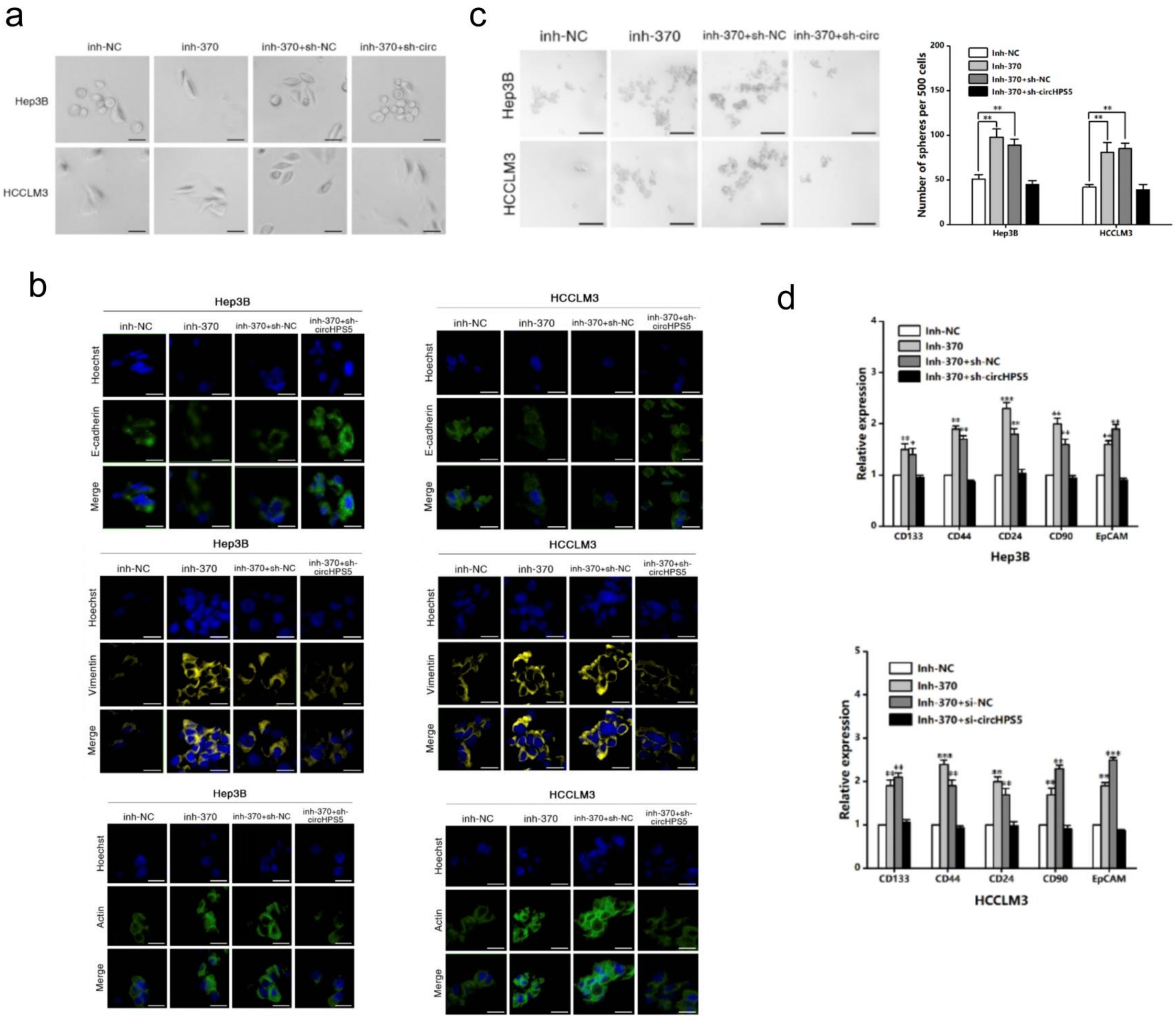
a
inh-NC
inh-370
inh-370+sh-NC
inh-370+sh-circ
Hep3B
HCCLM3
b
Hep3B
HCCLM3
inh-NC
inh-370
inh-370+sh-NC
inh-370+sh-circHPS5
Hoechst
E-cadherin
Merge
Vimentin
Actin
c
inh-NC
inh-370
inh-370+sh-NC
inh-370+sh-circ
Hep3B
HCCLM3
Inh-NC
Inh-370
Inh-370+sh-NC
Inh-370+sh-circHPS5
Number of spheres per 500 cells
d
Inh-NC
Inh-370
Inh-370+sh-NC
Inh-370+sh-circHPS5
Relative expression
CD133
CD44
CD24
CD90
EpCAM
Hep3B
Inh-NC
Inh-370
Inh-370+si-NC
Inh-370+si-circHPS5
Relative expression
CD133
CD44
CD24
CD90
EpCAM
HCCLM3
